# Mannose-binding lectin 2 gene polymorphisms and their association with tuberculosis in a Chinese population

**DOI:** 10.1186/s40249-020-00664-9

**Published:** 2020-04-29

**Authors:** Jun-Xian Zhang, Wen-Ping Gong, Dong-Lin Zhu, Hui-Ru An, You-Rong Yang, Yan Liang, Jie Wang, Jing Tang, Wei-guo Zhao, Xue-Qiong Wu

**Affiliations:** 1 Army Tuberculosis Prevention and Control Key Laboratory/Beijing Key Laboratory of New Techniques of Tuberculosis Diagnosis and Treatment, Institute for Tuberculosis Research, the 8th Medical Center of Chinese PLA General Hospital, 17# Heishanhu Road, Haidian District, Beijing, 100091 China; 2 Laboratory of Animal Experiment, the 8th Medical Center of Chinese PLA General Hospital, 17# Heishanhu Road, Haidian District, Beijing, 100091 China; 3 Physical Examination Center, the 8th Medical Center of Chinese PLA General Hospital, 17# Heishanhu Road, Haidian District, Beijing, 100091 China; 4 Department of Respiration, the 8th Medical Center of Chinese PLA General Hospital, 17# Heishanhu Road, Haidian District, Beijing, 100091 China

**Keywords:** Tuberculosis, Mannose-binding lectin, Single-nucleotide polymorphism, Genotype, Haplotype

## Abstract

**Background:**

Immune- and inflammation-related genes (IIRGs) play an important role in the pathogenesis of tuberculosis (TB). However, the relationship between IIRG polymorphisms and TB risk remains unknown. In this study, the gene polymorphisms and their association with tuberculosis were determined in a Chinese population.

**Methods:**

We performed a case-control study involving 1016 patients with TB and 507 healthy controls of Han Chinese origin. Sixty-four single-nucleotide polymorphisms (SNPs) belonging to 18 IIRGs were genotyped by the PCR-MassArray assay, and the obtained data was analyzed with *χ*^2^-test, Bonferroni correction, and unconditional logistic regression analysis.

**Results:**

We observed significant differences in the allele frequency of *LTA* rs2229094*C (*P* = 0.015), *MBL2* rs2099902*C (*P* = 0.001), *MBL2* rs930507*G (*P* = 0.004), *MBL2* rs10824793*G (*P* = 0.004), and *IL12RB1* rs2305740*G (*P* = 0.040) between the TB and healthy groups. Increased TB risk was identified in the rs930507 G/G genotype (*P*_*adjusted*_ = 0.027) under a codominant genetic model as well as in the rs2099902 (C/T + C/C) vs T/T genotype (*P*_*adjusted*_ = 0.020), rs930507 (C/G + G/G) vs C/C genotype (*P*_*adjusted*_ = 0.027), and rs10824793 (G/A + G/G) vs A/A genotype (*P*_*adjusted*_ = 0.017) under a dominant genetic model after Bonferroni correction in the analysis of the overall TB group rather than the TB subgroups. Furthermore, the rs10824793_rs7916582*GT and rs10824793_rs7916582*GC haplotypes were significantly associated with increased TB risk (*P* = 0.001, odds ratio [*OR*] = 1.421, 95% confidence interval [*CI*]: 1.152–1.753; and *P* = 0.018, *OR* = 1.364, 95% *CI*: 1.055–1.765, respectively). Moreover, the rs10824793_rs7916582*AT/AT or rs10824793_rs7916582*GT/GT diplotype showed a protective (*P* = 0.003, *OR* = 0.530, 95% *CI*: 0.349–0.805) or harmful (*P* = 0.009, *OR* = 1.396, 95% *CI*: 1.087–1.793) effect against the development of TB.

**Conclusions:**

This study indicated that *MBL2* polymorphisms, haplotypes, and diplotypes were associated with TB susceptibility in the Han Chinese population. Additionally, larger sample size studies are needed to further confirm these findings in the future.

## Background

Tuberculosis (TB) is a global infectious disease in humans. It is a severe and even lethal disease and was responsible for 1.2 million deaths worldwide in 2018 [1]. The main reason worldwide TB eradication is so difficult is that smear-positive TB patients are the most important source of infection. They often transmit the TB bacterium via droplets produced by coughing, sneezing, etc. It was found that a TB patient typically infects 10–15 people from the onset of the disease until diagnosis and treatment and that these infected people can, in turn, become new sources of infection. A healthy person’s chances of being infected with *Mycobacterium tuberculosis* depend on the number of droplets inhaled and duration as well as the individual’s immune status.

It is well known that approximately one-third of the world’s population is infected with *M. tuberculosis* [1], whereas only 10% of these infected individuals progress to TB disease [2]. This indicates that the risk of developing TB in humans is strongly associated with host-pathogen interactions, the environment, and genetic background [3]. Recently, a growing number of studies has supported the hypothesis that TB risk is associated with polymorphisms of immune- and inflammation-related genes (IIRGs), including the interleukin-10 (*IL-10*), *IL1A*, *IL1B, IL6*, *IL12B*, *IL27*, interleukin 12 receptor beta 1 (*IL12RB1*), interleukin 18 receptor 1 (*IL18R1*), signal transducer and activator of transcription 1 (*STAT1*), natural resistance-associated macrophage protein 1 (*SLC11A1* or *NRAMP1*), *SP110*, lymphotoxin A (*LTA*), tumor necrosis factor (*TNF*), interferon gamma receptor 1 (*IFNGR1*), *IFNGR2*, mannose-binding lectin 2 (*MBL2*), vitamin D receptor (*VDR*), monocyte chemoattractant protein-1 (*MCP-1* or *CCL2*), and toll-like receptor 8 (*TLR8*) genes [4–13]. IIRGs play essential roles in innate and adaptive immunity, which help control *M. tuberculosis* infection in humans [14]. Therefore, polymorphisms in these genes can alter immunity and lead to genetic susceptibility to TB. Moreover, common genetic variants of IIRGs could be used to predict and evaluate TB risk in the early stages of infection.

However, previous studies have mostly focused on the association between polymorphisms in one or several related genes and susceptibility to TB, rather than multiple IIRGs. It is well known that the interaction between *M. tuberculosis* and its host leads to a very complex immune response. As such, studies that focus on a single or a small number of sample genes may overlook potential associations between multiple genes: for example, they may ignore linkage disequilibrium between numerous single-nucleotide polymorphisms (SNPs). Therefore, it is imperative to study the association between various SNPs and susceptibility to TB in as many IIRGs as possible.

In this study, 64 SNPs in 18 IIRGs were selected, and the association between these SNPs and TB risk was evaluated using the polymerase chain reaction (PCR)-MassArray method in a large case-control population of Han Chinese origin.

## Methods

### Patients, controls, and ethics statement

This case-control study was performed in the 8th Medical Center of Chinese PLA General Hospital (Beijing, China) from June 2009 to March 2019 and was approved by the Research Ethics Committee of the 8th Medical Center of the Chinese PLA General Hospital. All DNA samples were extracted from residual blood after a liver function test. Informed consent was obtained from all participants. In total, 1016 patients (597 males and 419 females, mean age 39.5 ± 19.3 years) with a TB diagnosis according to smear, *M. tuberculosis* culture, radiological examination, and histological examination were randomly included from the patients in the 8th Medical Center of the Chinese PLA General Hospital (Beijing, China). In the same period, 507 healthy volunteers (289 males and 218 females, mean age 51.8 ± 10.6 years) with retrospectively confirmed non-tuberculous diseases were included from the physical examination center of 8th Medical Center of the Chinese PLA General Hospital. All TB and control patients were HIV negative.

### DNA extraction

Blood samples (2 ml) from each participant were collected (the residual portion of the blood samples obtained for a liver function test) and stored in citrate-anticoagulated glass tubes at − 40 °C until use. The Whole Blood DNA Extraction Kit (Tiangen Biotech, Co., Ltd., Beijing, China) was used to extract total genomic DNA from 1 ml of the stored blood samples, following the manufacturer’s instructions. Then, the extracted genomic DNA was resuspended in 0.1 × Tris-EDTA buffer (10 mmol/L Tris, 1 mmol/L EDTA, pH 8.0) and stored at − 20 °C.

### Screening of target SNPs

Data from the International HapMap Project (http://hapmap.ncbi.nlm.nih.gov) were used to screen potential SNPs using an estimated *r*^2^ threshold of > 0.8 for the untyped SNPs as reported in a previous study [15]. The genotype data for the Han Chinese population were obtained from the Haploview 4.2 program (http://www.broad.mit.edu/haploview) and used to select SNPs that have a minor allele frequency (MAF) of > 0.05.

### Genotyping

In total, 64 SNPs of *IL-10*, *IL18R1*, *IL1A*, *IL1B*, *STAT1*, *SLC11A1*, *SP110*, *IL12B*, *LTA*, *TNF*, *IFNGR1*, *MBL2*, *VDR*, *IL27*, *CCL2*, *IL12RB1*, *IFNGR2*, and *TLR8* were genotyped in samples from both TB patients and controls using the iPLEX assay on a MassArray system (Sequenom Inc., San Diego, United States) according to a previously published protocol [16]. The main particularities are listed below: (1) PCR reactions: Genomic DNA (10 ng), *Taq* DNA polymerase (0.5 U, HotStarTaq, Qiagen, Shanghai, China), dNTPs (500 μmol), and PCR primers (100 nmol) in a 5-μl reaction volume were added into a 384-well plate. Then, PCR thermal cycling was performed at 94 °C, followed by 45 cycles of 20 s at 94 °C, 30 s at 56 °C, and 60 s at 72 °C using an ABI-9700 instrument (Thermo Fisher Scientific Inc., Waltham, United States). Finally, the PCR products were examined by 2.0% agarose gel electrophoresis. (2) Purification: After the PCR reaction, 2 μl of shrimp alkaline phosphatase (0.3 U) was mixed with the PCR products, incubated at 37 °C for 20 min, and then inactivated at 85 °C for 5 min. (3) Extension: The concentrations of the extension primers were adjusted to equilibrate the signal-to-noise ratios. Then, termination mix (100 μmol), DNA polymerase (0.05 U, Sequenom, Inc., San Diego, United States), and extension primers (625 to 1250 nmol/L) in a final volume of 9 μl were pooled together and detected using an iPLEX Gold Kit (Sequenom, Inc., San Diego, United States) at 94 °C for 30 s, followed by 5 s at 94 °C and 5 cycles of 5 s at 52 °C and 5 s at 80 °C. An additional 40 annealing and extension cycles were then performed, with 5 s at 94 °C and 5 cycles of 5 s at 52 °C and 5 s at 80 °C. The final extension was carried out at 72 °C for 3 min; then, the sample was cooled to 4 °C. (4) MALDI-TOF-MS: The samples were then manually desalted using 6 mg of clean resin and a dimple plate and subsequently transferred to a 384-well Spectro-CHIP (Sequenom, Inc., San Diego, United States) using a nano-dispenser. The mass spectra were acquired using the Compact Mass Spectrometer and analyzed via the MassArray Typer 4.0 Software (Sequenom, Inc., San Diego, United States). The PCR assay was performed with two no-template controls and four duplicated samples in each 384-well format as quality controls. Each genotyping result was generated and analyzed by laboratory staff who were unaware of the patient’s status.

### Statistical analyses

All statistical analyses were performed using the Stata statistical package (version 10.0; StataCorp LP, College Station, TX, USA), and all *P* values were two-tailed. The *statistical* differences in allele and genotype frequencies between the TB and control groups were evaluated using the *χ*^2^-test. In the *χ*^2^-test, *P* values with a Bonferroni correction of < 0.05 were considered significant. The Hardy-Weinberg Equilibrium (HWE) was tested via the *χ*^2^-test for goodness of fit using a web program (http://ihg.gsf.de/cgi-bin/hw/hwa1.pl). Moreover, Akaike’s information criterion was used to select the genetic model with maximum parsimony for each SNP. Odds ratios (*OR*s) as well as 95% confidence intervals (*CI*s) were calculated via unconditional logistic regression analysis with adjustment for age and gender.

The pairwise linkage disequilibrium (LD) among the SNPs was determined using Lewontin’s standardized coefficient D’ and LD coefficient *r*^2^ as described in a previous study [17], whereas haplotype blocks were defined in Haploview 4.2 (https://www.broadinstitute.org/haploview/haploview) with default settings following the criteria published in a previous study [18]. In addition, the haplotype frequencies were estimated using the PHASE 2.1 Bayesian algorithm [19] and HAPLO.STATS [20]. The haplotypes were then pooled into a combined group if their frequency was less than 0.03. Empirical *P* values, based on 100 000 simulations, were computed for the global score test and each of the haplotype-specific score tests. The diplotype (haplotype dosage, an estimate of the number of copies of the haplotype) was the most probable haplotype pair for each individual. Unconditional logistic regression analysis was used to evaluate the *OR*s and 95% *CI*s for participants carrying 1 to 2 copies versus 0 copies of each common haplotype for the dichotomized diplotypes.

## Results

### The distribution of 64 SNP alleles in TB patients and healthy controls

One thousand and sixteen patients with a TB diagnosis and 507 healthy controls were recruited. Among the TB patients, 680 (66.9%) had total pulmonary TB (TPTB), including 388 with simple PTB and 74 with simple TB pleurisy (TBP), 166 (16.3%) had extrapulmonary TB (EPTB), and 170 (16.7%) had concomitant PTB and EPTB (PTB + EPTB).

Sixty-four SNPs from 18 IIRGs were selected and genotyped, and all allele distributions in the control group were consistent with those from the HWE (*P* > 0.01, Table 1). The results showed that the allele distributions of *LTA* rs2229094*C (*P* = 0.015), *MBL2* rs2099902*C (*P* = 0.001), *MBL2* rs930507*G (*P* = 0.004), *MBL2* rs10824793*G (*P* = 0.004), and *IL12RB1* rs2305740*G (*P* = 0.040) were significantly different between the TB patients and healthy controls (Table 1), whereas the allele distributions of the other SNPs were not.

**Table 1:** Information about 64 genotyped SNPs in the *IL-10, IL18R1, IL1A, IL1B, STAT1, SLC11A1, SP110, IL12B, LTA, TNF, IFNGR1, MBL2, VDR, IL27, CCL2, IL12RB1, IFNGR2,* and TLR8

Gene: locus and OMIM No.^a^	No.	SNP_ID	Chromosome No.	Chromosome position ^b^	Intermarker distances ^c^ (bp)	Genic location	Base Change	MAF ^d^			*P*^f^	*P* value for HWE ^g^ test	Genotyping rate ^h^(%)
								NCBI ^e^	Control	Tuberculosis			
IL10: 1q31-q32 OMIN: 124092	1	rs3024496	1	206 768 519	–	3’UTR	T → C	0.0097	0.051	0.049	0.730	0.5467	99.7
	2	rs1800871	1	206 773 289	4770	Intron	T → C	0.2573	0.364	0.341	0.226	0.5098	98.7
	3	rs1800896	1	206 773 552	263	Intron	A → G	0.0340	0.092	0.092	0.964	0.8843	100
IL18R1: 2q12 OMIN: 604494	1	rs3771167	2	102 369 728	–	Intron	T → C	0.0485	0.035	0.035	0.902	0.4203	100
	2	rs1974675	2	102 369 915	187	Intron	C → T	0.1456	0.136	0.126	0.435	0.8773	99.8
	3	rs6758936	2	102 374 909	4994	Intron	G → A	0.1699	0.142	0.141	0.944	0.6809	99.8
	4	rs6750020	2	102 378 254	3345	Intron	G → A	0.4806	0.459	0.442	0.376	0.1350	99.6
	5	rs1035130	2	102 384 942	6688	Exon 6 F251F	G → A	0.3495	0.317	0.299	0.301	0.1045	99.5
	6	rs3771158	2	102 393 434	8492	Intron	T → C	0.0971	0.097	0.088	0.439	0.7258	99.7
IL1A: 2q14 OMIN: 147760	1	rs17561	2	112 779 646	–	Exon 4 A114S	G → T	0.0631	0.121	0.098	0.063	0.8821	99.6
	2	rs3783526	2	112 784 230	4584	Intron	A → G	0.3689	0.351	0.367	0.374	0.0129	99.3
IL1B: 2q14 OMIN: 147720	1	rs2853550	2	112 829 544	45 314	Intron	C → T	0.0825	0.111	0.112	0.913	0.1487	99.9
	2	rs1143633	2	112 832 890	3346	Intron	A → G	0.4126	0.435	0.426	0.658	0.5667	98.9
	3	rs1143627	2	112 836 810	3920	UTR-5	T → C	0.4563	0.497	0.489	0.685	0.7878	97.0
STAT1: 2q32.2 OMIN: 600555	1	rs2280235	2	190 979 104	–	Intron	C → T	0.4660	0.484	0.465	0.335	0.2941	98.7
	2	rs16833155	2	190 996 651	17 547	Intron	C → T	0.0291	0.053	0.054	0.982	0.6981	99.9
	3	rs13029247	2	191 001 932	5281	Intron	C → T	0.5000	0.476	0.462	0.463	0.0728	98.8
	4	rs7576984	2	191 003 857	1925	Intron	C → A	0.1650	0.167	0.160	0.647	0.2140	99.7
	5	rs2066802	2	191 009 941	6084	Exon 1 L21L	T → C	0.2427	0.218	0.210	0.620	0.2973	99.2
SLC11A1: 2q35 OMIN: 600266	1	rs2276631	2	218 384 290	–	Exon 2 F66F	G → A	0.1408	0.144	0.149	0.665	0.3834	99.2
	2	rs17221959	2	218 387 907	3617	Exon 7 G249G	C → T	0.1068	0.099	0.112	0.295	0.9842	95.4
	3	rs17235409	2	218 395 009	7102	Exon 14 D543N	G → A	0.1408	0.138	0.125	0.311	0.5529	99.1
SP110: 2q37.1 OMIN: 604457	1	rs9783992	2	230 170 873	–	Intron	T → C	0.0049	0.002	0.002	0.989	0.9645	99.9
	2	rs10165685	2	230 174 882	4009	Intron	G → A	0.1262	0.159	0.157	0.845	0.6981	99.8
	3	rs957683	2	230 178 660	3778	Intron	T → C	0.3738	0.456	0.445	0.566	0.1516	98.7
	4	rs41345344	2	230 200 212	21 552	Intron	C → G	0.2718	0.170	0.185	0.322	0.4374	98.5
	5	rs1365776	2	230 207 994	7781	Exon 7 G305R	A → G	0.1068	0.126	0.114	0.351	0.4184	99.6
IL12B: 5q31.1-q33.1 OMIN: 161561	1	rs1368439	5	159 315 006	–	3’URT	T → G	0.0097	0.004	0.003	0.496	0.9289	99.8
	2	rs919766	5	159 320 556	5550	Intron	A → C	0.0437	0.052	0.053	0.901	0.7283	100
	3	rs3212217	5	159 328 122	7566	Intron	G → C	0.4417	0.455	0.427	0.145	0.1761	99.6
	4	rs2546892	5	159 328 467	345	Intron	G → A	0.1845	0.186	0.212	0.101	0.8839	96.5
LTA: 6p21.3 OMIN: 153440	1	rs2009658	6	31 570 467	–	Intron	C → G	0.1845	0.144	0.171	0.057	0.3741	99.4
	2	rs1800683	6	31 572 294	1827	5’UTR	G → A	0.4175	0.438	0.420	0.342	0.5831	98.7
	3	rs2229094	6	31 572 779	485	Exon 1 C13R	T → C	0.2282	0.190	0.229	**0.015**	0.1732	99.3
	4	rs2229092	6	31 572 980	201	Exon 2 H51P	A → C	0.0243	0.021	0.024	0.631	0.0871	99.9
	5	rs1041981	6	31 573 007	27	Exon 2 T60N	C → A	0.4175	0.437	0.419	0.349	0.6545	99.0
TNF: 6p21.3 OMIN: 191160	1	rs1800629	6	31 575 254	2247	Intron	G → A	0.0922	0.054	0.069	0.100	0.1735	99.5
	2	rs3093662	6	31 576 412	1158	Intron	A → G	0.0340	0.038	0.048	0.183	0.7276	99.3
IFNGR1: 6q23.3 OMIN: 107470	1	rs1887415	6	137 198 101	–	Exon 7 L467P	T → C	0.0194	0.029	0.031	0.743	0.5056	99.7
	2	rs2234711	6	137 219 383	21 282	5’UTR	C → T	0.4757	0.453	0.448	0.811	0.2336	99.3
MBL2: 10q11.2 OMIN: 154545	1	rs2099902	10	52 766 089	–	3’UTR	T → C	0.2670	0.198	0.255	**0.001**	0.4386	99.3
	2	rs930507	10	52 768 506	2417	Exon 4 L126L	C → G	0.2427	0.191	0.237	**0.004**	0.8992	97.1
	3	rs10824793	10	52 769 720	1214	Intron	A → G	0.3398	0.285	0.336	**0.004**	0.9966	99.9
	4	rs7916582	10	52 773 235	3515	Intron	T → C	0.1456	0.111	0.126	0.232	0.6949	99.6
VDR: 12q13.11 OMIN: 601769	1	rs2239184	12	47 850 800	–	Intron	C → T	0.2913	0.279	0.271	0.637	0.8753	99.8
	2	rs2248098	12	47 859 573	8773	Intron	T → C	0.2621	0.292	0.281	0.519	0.2028	99.7
	3	rs1540339	12	47 863 543	3970	Intron	A → G	0.2670	0.289	0.316	0.124	0.3715	99.7
	4	rs10783219	12	47 901 705	38 162	Intron	A → T	0.4175	0.433	0.465	0.110	0.4119	94.3
	5	rs7139166	12	47 906 551	4846	Intron	C → G	0.0243	0.028	0.027	0.852	0.5221	99.9
IL27: 16p11 OMIN: 608273	1	rs181206	16	28 502 082	–	Exon 4 L119R	T → C	0.1456	0.136	0.129	0.621	0.3844	99.0
CCL2: 17q11.2-q12 OMIN: 158105	1	rs4586	16	34 256 250	–	Exon 2 C35C	C → T	0.3641	0.412	0.380	0.092	0.9518	99.1
IL12RB1: 19p13.1 OMIN: 601604	1	rs2305740	19	18 069 426	–	Intron	A → G	0.0922	0.136	0.110	**0.040**	0.7880	99.9
	2	rs401502	19	18 069 603	177	Exon 11 G378R	C → G	0.3350	0.369	0.344	0.186	0.5334	99.8
	3	rs375947	19	18 069 641	38	Exon 11 M365T	A → G	0.3350	0.368	0.342	0.164	0.4755	99.5
	4	rs17852635	19	18 075 765	6124	Exon 7 P228P	G → A	0.3350	0.368	0.332	0.055	0.6152	99.1
	5	rs11575934	19	18 075 808	43	Exon 7 Q214R	A → G	0.3350	0.375	0.339	0.051	0.9668	98.8
IFNGR2: 21q22.11 OMIN: 147569	1	rs1059293	21	33 437 386	–	3’URT	T → C	0.1553	0.121	0.114	0.578	0.0179	99.5
TLR8: Xp22 OMIN: 300366	1	rs3764880	X	12 906 707	–	Exon 1 M1V	G → A	0.1778	0.153	0.155	0.917	–	99.5
	2	rs5744068	X	12 916 939	10 232	Intron	C → T	0.2250	0.017	0.019	0.667	–	99.9
	3	rs2159377	X	12 919 394	2455	Exon 2 D136D	T → C	0.2813	0.210	0.205	0.771	–	99.3
	4	rs5744080	X	12 919 685	291	Exon 2 H233Q	T → C	0.2313	0.194	0.184	0.504	–	99.7
	5	rs2407992	X	12 920 993	1308	Exon 2 L669L	C → G	0.2250	0.190	0.177	0.379	–	99.1
	6	rs3747414	X	12 921 293	300	Exon 2 I769I	A → C	0.3221	0.196	0.177	0.225	–	99.0
	7	rs5744088	X	12 922 445	1152	3’URT	G → C	0.0500	0.025	0.024	0.958	–	98.9

^a^OMIM, Online Mendelian Inheritance in Man (http://www.ncbi.nlm.nih.gov/Omim)

^b^SNP position in the NCBI dbSNP database (http://www.ncbi.nlm.nih.gov/SNP)

^c^Intermarker distances, the distance between two adjacent SNP sites on the same gene sequence. The intermarker distance of first SNP was showed as “——”

^d^*MAF*, minor allele frequency

^e^MAF for Chinese in the NCBI dbSNPs database

^f^*P* value for difference in allele distributions between tuberculosis and control group

^g^*HWE* Hardy-Weinberg equilibrium in the control group

^h^Genotyping Rate, rate of actual genotyping samples to total samples

-: Not applicable

### The genotypic frequencies of SNPs and their associations with TB risk

When investigating the TB group, the unconditional logistic regression analysis showed that 14 SNPs of *IL18R1*, *IL1A*, *STAT1*, *LTA*, *IFNGR1*, *MBL2*, *VDR*, and *IL12RB1* were associated with TB risk under a codominant model (Table S1) and under a dominant and recessive genetic model (Table S2). However, after adjusting for the Bonferroni correction, only SNPs in the *MBL2* gene were found to still be associated with TB risk. Therefore, we next focused on the *MBL2* gene. Our results showed that: 1) Under a codominant genetic model (Table 2), the rs2099902 C/T and C/C genotypes, rs930507 C/G genotype, rs10824793 G/A and G/G genotypes, and rs7916582 T/C genotype were associated with increased risk of TB. After the Bonferroni correction, increased TB risk was still observed in patients with a rs930507 G/G genotype (*P*_*adjusted*_ = 0.027). 2) Under a dominant and recessive genetic model (Table 3), the rs2099902 (C/T + C/C) vs T/T and C/C vs (T/T + C/T) genotypes, rs930507 (C/G + G/G) vs C/C genotype, rs10824793 (G/A + G/G) vs A/A as well as G/G vs (A/A + G/A) genotypes, and rs7916582 (T/C + C/C) vs T/T genotype were associated with increased risk of TB. Interestingly, increased TB risk was still observed for the rs2099902 (*P*_*adjusted*_ = 0.020), rs930507 (*P*_*adjusted*_ = 0.027), and rs10824793 (*P*_*adjusted*_ = 0.017) SNPs under a dominant genetic model after the Bonferroni correction.

**Table 2:** Genotype frequencies of SNPs in the *MBL2* gene among cases and controls and their associations with tuberculosis risk under a codominant genetic model

SNP ID	Genotype	Case (TB)		Control		*P* (2 df)^a^	Logistic Regression		
		No.	Frequency	No.	Frequency		*OR* (95% *CI*)	*P*^b^	*P*_*adjusted*_^c^
rs2099902	T/T	552	55.8%	323	63.8%	0.0025	1.000 (referent)		
	C/T	372	37.6%	166	32.8%		2.364 (1.317–4.244)	**0.004**	0.256
	C/C	66	6.7%	17	3.4%		1.459 (1.143–1.863)	**0.002**	0.128
**rs930507**	C/C	549	57.3%	330	65.4%	0.0149	1.000 (referent)		
	C/G	363	37.9%	157	31.1%		**1.556 (1.215–1.992)**	**4.218E-4**	**0.027**
	G/G	46	4.8%	18	3.6%		1.672 (0.921–3.038)	0.091	1.000
rs10824793	A/A	434	43.4%	259	51.2%	0.0171	1.000 (referent)		
	G/A	459	46.0%	206	40.7%		1.466 (1.153–1.863)	**0.002**	0.128
	G/G	106	10.6%	41	8.1%		1.890 (1.245–2.870)	**0.003**	0.192
rs7916582	T/T	762	76.3%	398	79.3%	0.3794	1.000 (referent)		
	T/C	223	22.3%	97	19.3%		1.338 (1.006–1.779)	**0.045**	1.000
	C/C	14	1.4%	7	1.4%		1.134 (0.424–3.034)	0.803	1.000

^a^Global *P* values (2 degrees of freedom [df]): genotype frequencies in tuberculosis and control group were compared using a *χ*^2^ test with two df

^b^*P* values from unconditional logistic regression analyses, adjusted for age and gender

^c^*P*_*adjusted*_, *P* value with Bonferroni correction, *P*_*adjusted*_ value less than 0.05 was considered to be significant

**Table 3:** Association analysis of SNPs in the *MBL2* gene under a dominant and recessive genetic model

SNP ID	Genetic model	Case	Control	Logistic Regression		
				*OR* (95% *CI*)	*P*^a^	*P*_*adjusted*_^b^
rs2099902	(C/T + C/C) vs T/T	438/552	183/323	1.544 (1.220–1.954)	**3.023E-3**	**0.020**
	C/C vs (T/T + C/T)	66/924	17/489	2.055 (1.154–3.659)	**0.014**	0.896
rs930507	(C/G + G/G) vs C/C	409/549	175/330	1.568 (1.235–1.990)	**2.211E-4**	**0.027**
	G/G vs (C/C + C/G)	46/912	18/487	1.425 (0.790–2.568)	0.239	1.000
rs10824793	(G/A + G/G) vs A/A	565/434	247/259	1.533 (1.219–1.927)	**2.544E-4**	**0.017**
	G/G vs (A/A + G/A)	106/893	41/465	1.571 (1.052–2.345)	**0.027**	1.000
rs7916582	(T/C + C/C) vs T/T	237/762	104/398	1.324 (1.003–1.748)	**0.047**	1.000
	C/C vs (T/T + T/C)	14/985	7/495	1.065 (0.399–2.841)	0.900	1.000

^a^*P* values from unconditional logistic regression analyses, adjusted for age and gender

^b^*P*_*adjusted*_*P* value with Bonferroni correction, the *P*_*adjusted*_ value less than 0.05 was considered to be significant

### The distribution of the *MBL2* SNP genotype frequency

To further confirm the differences in the distribution of the *MBL2* SNP genotype frequency between the TB subgroups (TPTB, PTB, EPTB, and PTB + EPTB) and healthy controls, we performed unconditional logistic regression analysis under codominant, dominant, and recessive genetic models. The results indicated that the rs2099902 C/T and C/C genotypes, rs930507 C/G genotype, rs10824793 G/A and G/G genotypes were associated with increased TB risk in the TB subgroups (Table S3). However, these statistically significant differences disappeared after applying the Bonferroni correction under a codominant genetic model (*P*_*adjusted*_ > 0.05, Table S3). A similar result was observed under the dominant and recessive genetic model (*P*_*adjusted*_ > 0.05, Table S4).

### The distribution of the *MBL2* haplotypes and diplotypes

**Fig. 1:**
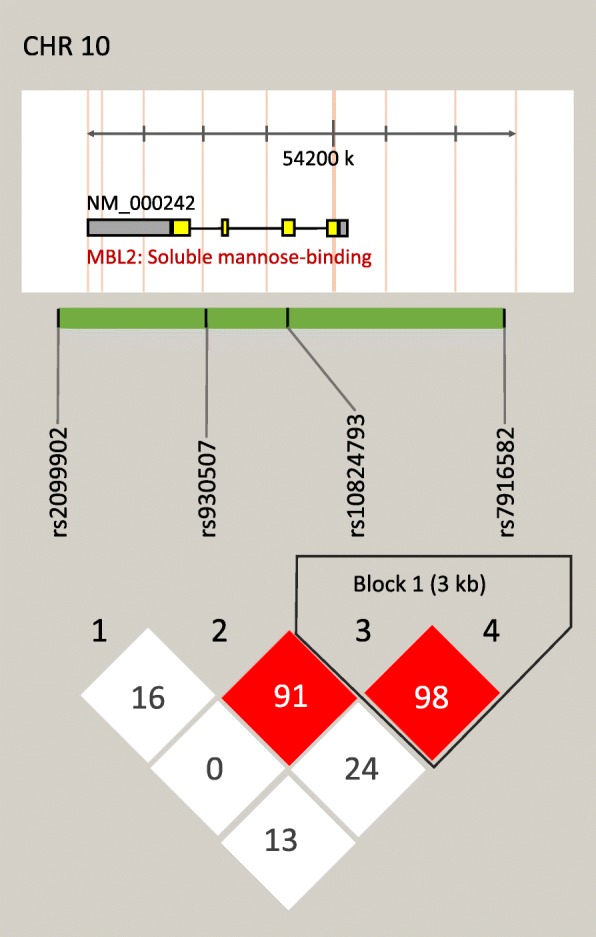
Location and linkage disequilibrium structure for the four SNPs in the *MBL2* gene. The SNP distribution and haplotype block structure across four SNPs in the *MBL2* gene are shown, respectively. The figure was composed of chromosome-scale (the top line with even division), the transcription string (the thick bar represents exon (yellow), or UTR (grey), and the thin line represent intron), SNP scale (the hollow bar with scales representing SNPs location), and graphic of LD (black-and-green) or block definition (flammulated). Correlation coefficients (*r*^2^, × 100) are shown in the individual boxes; the color from white to red denotes *r*^2^ from 0 to 1. LD, linkage disequilibrium; UTR, Untranslated Regions

To investigate the associations regarding LD patterns between these four SNPs, we used Haploview to plot their haplotype blocks. We identified one haplotype block composed of rs10824793 and rs7916582 (*r*^2^ = 0.98). However, the rs2099902 and rs930507 SNPs in the *MBL2* gene were outside this haplotype block (Fig. 1). In the haplotype analysis, three common haplotypes (rs10824793_rs7916582*AT, GT, and GC) were observed among the participants; the total percentage of these common haplotypes was as high as 99.85% in the TB group or 99.9% in the control group. Conversely, the total percentage of other haplotypes was only 0.15% or 0.10% in the TB or control group, respectively (Table 4). The global score test indicated that the frequency of the haplotypes from the block between the TB and control groups was significantly different (global *P* = 0.00222, *P*_sim_ = 0.00207). Interestingly, statistical differences were observed in the frequency of the rs10824793_rs7916582*AT (*P* = 0.00014) or rs10824793_rs7916582*GT (*P* = 0.003) haplotype between the TB and control groups. Moreover, this difference remained significant after the Bonferroni correction (rs10824793_rs7916582*AT, *P*_*adjusted*_ = 0.00042; rs10824793_rs7916582*GT, *P*_*adjusted*_ = 0.009). Furthermore, the rs10824793_rs7916582*GT or rs10824793_rs7916582*GC haplotype was significantly associated with increased TB risk (*P* = 0.001, *OR*: 1.421, 95% *CI*: 1.152–1.753; or *P* = 0.018, *OR*: 1.364, 95% *CI*: 1.055–1.765) in the logistic regression analysis when compared to the rs10824793_rs7916582*AT haplotype (Table 4).

**Table 4:** Haplotypes analysis of *MBL2* gene polymorphisms with tuberculosis risk

Haplotype	Tuberculosis		Control		*P*^a^	*P*_*adjusted*_^b^	Hap. Score^c^	Logistic Regression		Global score test
	No.	Frequency	No.	Frequency				*OR* (95% *CI*)	*P*^d^	
Block 1: **rs10824793-rs7916582**										Global-stat = 14.57143, df ^e^ = 3, *P* = 0.00222, P_sim_ = 0.00207
AT	1328	66.3%	723	71.4%	**0.00014**	**0.00042**	−3.8039	1.000 (referent)		
GT	423	21.1%	177	17.5%	**0.003**	**0.009**	2.9843	**1.421 (1.152–1.753)**	**0.001**	
GC	248	12.4%	111	11.0%	0.077	0.231	1.7679	**1.364 (1.055–1.765)**	**0.018**	
Others	3	0.2%	1	0.1%	–	–	–	–	–	

^a^*P* value for difference in the haplotype frequency between tuberculosis and control group

^b^*P*_*adjusted*_, *P* value with Bonferroni correction, *P*_*adjusted*_ value less than 0.05 was considered to be significant

^c^A positive (or negative) score for a particular haplotype would have suggested that the haplotype was associated with increased (or decreased) risk of Tuberculosis

^d^*P* values from unconditional logistic regression analyses, adjusted for age and gender

^e^*df* degrees of freedom

Moreover, the association between the diplotypes of the *MBL2* gene polymorphisms and TB risk was also analyzed. As shown in Table 5, the diplotype composed of the rs10824793_rs7916582*AT haplotypes had a considerably decreased TB risk in a 2-copy logistic regression analysis compared with 0-copy (*P* = 0.003, *OR* = 0.530, 95% *CI*: 0.349–0.805). Moreover, this significant protective effect was still observed after Bonferroni correction (*P*_*adjusted*_ = 0.009). In contrast, increased TB risk was found in the diplotype composed of the rs10824793_rs7916582*GT (*P* = 0.009, *OR* = 1.396, 95% *CI*: 1.087–1.793) or rs10824793_rs7916582*GC (*P* = 0.05, *OR* = 1.330, 95% *CI*: 1.000–1.768) haplotypes in 1-copy logistic regression analysis compared with 0-copy. However, this significant difference was only observed in the diplotype composed of the rs10824793_rs7916582*GT haplotype after Bonferroni correction (*P*_*adjusted*_ = 0.027).

**Table 5:** Diplotypes analysis of *MBL2* gene polymorphisms with tuberculosis risk

Haplotype	0-copy		1-copy Logistic Regression				2-copy Logistic Regression			
	Case/Control	*OR* (95% *CI*)	Case/Control	*P*^a^	*P*_*adjusted*_^b^	*OR* (95% *CI*)	case/control	*P*^a^	*P*_*adjusted*_^b^	*OR* (95% *CI*)
rs10824793_rs7916582*AT	106/41	1.000 (referent)	462/207	0.235	0.705	0.775 (0.509–1.180)	433/258	**0.003**	**0.009**	**0.530 (0.349**–**0.805)**
rs10824793_rs7916582*GT	622/345	1.000 (referent)	335/145	**0.009**	**0.027**	**1.396 (1.087**–**1.793)**	44/16	0.101	0.303	1.684 (0.904–3.136)
rs10824793_rs7916582*GC	767/402	1.000 (referent)	220/97	**0.05**	0.15	**1.330 (1.000**–**1.768)**	14/7	0.797	2.391	1.138 (0.426–3.041)

^a^*P* values from unconditional logistic regression analyses, adjusted for age and gender

^b^*P*_*adjusted*_, *P* value with Bonferroni correction, *P*_*adjusted*_ value less than 0.05 was considered to be significant

## Discussion

**Fig. 2:**
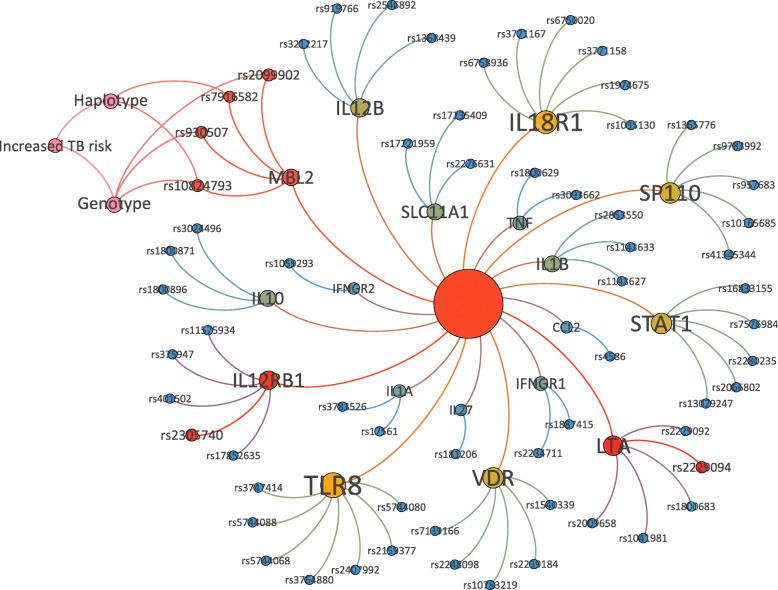
Neural network diagram of 64 SNPs in the 18 immune- and inflammation-related genes (IIRGs). The neural network diagram was plotted using an open-source graph visualization and manipulation software termed Gehpi. In the present figure, 18 genes and their SNPs were represented by solid dots. The circle size and color of the dot represent the number of connection degree, red represents the maximum connection degree, and blue represents the minimum connection degree. Three genes (*MBL2*, *LTA*, and *IL12RB1*) and their significant SNPs were showed as red color

In this study, we genotyped 64 SNPs from 18 IIRGs in a Han Chinese population. We first showed that the rs930507 G/G, rs2099902 [(C/T + C/C) vs T/T], rs930507 [(C/G + G/G) vs C/C], and rs10824793 [(G/A + G/G) vs A/A] genotypes were risk factors for TB under a codominant or dominant genetic model in TB patients and healthy controls (Fig. 2). Interestingly, these significant associations were not observed under any genetic model between subgroups of the TB patients and controls. This may be attributed to the low number of patients included in each tuberculosis subgroup. Therefore, to further improve the accuracy of the study, the sample size of each tuberculosis subgroup should be increased in the future.

The MBL protein is encoded by the *MBL2* gene and is secreted in the liver, where it activates the complement system via the lectin pathway to combat pathogens during host infection [21]. Although the mechanisms by which the *MBL2* mutations regulate TB progression remain unclear, there is no doubt that *MBL2* plays a vital role in the pathophysiology of TB. To our knowledge, this is the first study to report that the rs930507, rs2099902, and rs10824793 polymorphisms can affect TB development in a population of Han Chinese origin. It is worth mentioning that several meta-analysis studies have reported that five *MBL2* SNPs (rs1800450, rs1800451, rs5030737, rs7095891, and rs7096206) are associated with an increased or decreased TB risk [22–26]. However, there is insufficient data regarding the role of rs930507, rs2099902, and rs10824793 in TB susceptibility. Several studies on diseases other than TB have revealed that rs930507 was associated with an increased risk of invasive pneumococcal disease (IPD) in African Americans [27] and otitis media in children younger than 2 years of age [28]. Moreover, it was also shown to be associated with sodium-lithium countertransport (SLC) and systolic blood pressure [29]. Previous studies have found no association between rs2099902 and recurrent vulvovaginal infections risk [30] or severe dengue [31]; however, another study performed by Zanetti et al. suggested that rs2099902 was associated with increased risk of colon cancer in African Americans [32]. These data indicate that the susceptibility and pathogenicity of the same SNP were different in various diseases. As such, the mechanisms that underlie these differences might deserve further investigation.

The above-mentioned evidence indicated an association between the *MBL2* gene and TB risk genotypes. Herein, we found an association between them by linkage disequilibrium, haplotype, and diplotype analyses. In this study (Fig. 2), the rs7916582 polymorphism was not found to be significantly associated with TB susceptibility. However, when the rs10824793 and rs7916582 SNPs were combined in haplotypes, the rs10824793*G/rs7916582*T and rs10824793*G/rs7916582*C alleles were found to be significantly associated with TB risk, which is similar to the haplotype block rs7095891*G/rs1800450*C/rs1800451*C/rs4935047*A/rs930509*G/rs2120131*G/rs2099902*C yielded by LD analysis in a previous study [31]. LD is the non-random combination of alleles at different loci and is influenced by several factors, such as selection, genetic drift, recombination rate, mutation rate, and population structure as well as genetic linkage. A haplotype is a group of genes in an organism that are inherited together from a single parent. Haplotypes are critical for investigating the genetics of common diseases, which have been studied in humans through the International HapMap Project [33].

Analyses of polymorphism data based on LD and haplotype structure are becoming increasingly important; both have been successfully used to determine the association between *MBL2* polymorphisms and TB susceptibility. A previous study indicated that *MBL2* gene diplotypes might be significantly more common in TB patients than in the control group [24]. It is well known that the haplotype or genotype information can be statistically defined as complete or incomplete data because the genotype data can be extracted from the haplotype data, but the reverse is not true. Consequently, it seems more important to determine the association between polymorphism and phenotype based on the configuration of haplotypes and diplotypes compared with alleles and genotypes. Recently, some studies have indicated that reactions to drugs and phenotypes are associated with the arrangement of haplotypes or diplotype rather than genotypes [34], which is consistent with the results of our present study. Although there were no significant differences in the *MBL2* alleles observed between the TB and control groups, haplotype or diplotype configuration analysis found that the rs10824793_rs7916582*AT/AT diplotype had a significantly decreased TB risk in 1-copy logistic regression analysis compared with 0-copy, but the rs10824793_rs7916582*GT/GT diplotype had a considerably increased TB risk.

However, the limitation of the present study is that we did not analyze the relationship between MBL levels and TB risk. It has been reported that serum MBL levels were significantly higher in patients with active TB than in healthy controls [35], which may protect against the early development of pulmonary TB after infection [36].

## Conclusions

This case-control study showed, for the first time, that the rs930507 G/G, rs2099902 (C/T + C/C) vs T/T, rs930507 (C/G + G/G) vs C/C, and rs10824793 (G/A + G/G) vs A/A genotypes were associated with an increased risk of TB in the Han Chinese population. Interestingly, our findings also showed that the rs10824793_rs7916582*GT and rs10824793_rs7916582*AT haplotypes or diplotypes were significantly associated with TB risk. These findings provide new insights into the association between SNPs in IIRGs and susceptibility to TB. However, it is necessary to confirm the findings of our study by performing further multi-centric clinical and extensive sample studies on different populations in China.

## Supplementary information

**Additional file 1: Table S1.** Genotype frequencies of 64 genotyped SNPs in the *IL-10*, *IL18R1*, *IL1A*, *IL1B*, *STAT1*, *SLC11A1*, *SP110*, *IL12B*, *LTA*, *TNF*, *IFNGR1*, *MBL2*, *VDR*, *IL27*, *CCL2*, *IL12RB1*, *IFNGR2* and *TLR8* genes among cases and controls and their associations with tuberculosis risk under a codominant genetic model.

**Additional file 2: Table S2.**. Association analysis of 64 SNPs in the *IL-10*, *IL18R1*, *IL1A*, *IL1B*, *STAT1*, *SLC11A1*, *SP110*, *IL12B*, *LTA*, *TNF*, *IFNGR1*, *MBL2*, *VDR*, *IL27*, *CCL2*, *IL12RB1*, *IFNGR2* and *TLR8* genes under a dominant and recessive genetic model.

**Additional file 3: Table S3.** Association analysis of *MBL2* SNPs between TB subgroups and healthy controls under a codominant genetic model.

**Additional file 4: Table S4.** Association analysis of *MBL2* SNPs between TB subgroups and healthy controls under a dominant and recessive genetic model.

## Data Availability

All data generated or analyzed during this study are included in this published article [and its supplementary information files].

## Abbreviations

*CI*s: Confidence intervals
EPTB: Extrapulmonary tuberculosis
HWE: Hardy-Weinberg Equilibrium
IFNGR1: Interferon gamma receptor 1
IL-10: Interleukin-10
IL12RB1: Interleukin 12 receptor beta 1
IL18R1: Interleukin 18 receptor 1
IIRGs: Immune- and Inflammatory-Related Genes
LD: Linkage Disequilibrium
LTA: Lymphotoxin A
MAF: Minor Allele Frequency
MBL: Mannose-Binding Lectin
MCP-1 or CCL2: Monocyte chemoattractant protein-1
NRAMP1 or SLC11A1: Natural resistance-associated macrophage protein 1
*OR*s: Odds ratios
PCR: Polymerase Chain Reaction
PTB: Pulmonary Tuberculosis
SLC: Sodium-Lithium Countertransport
SNPs: Single-Nucleotide Polymorphisms
STAT1: Signal transducer and activator of transcription 1
TB: Tuberculosis
TLR8: Toll-like receptor 8
TNF: Tumor necrosis factor
TPTB: Total pulmonary tuberculosis
VDR: Vitamin D receptor
