# Supplementary material for: Mannose-binding lectin 2 gene polymorphisms and their association with tuberculosis in a Chinese population
Source: Infect Dis Poverty. 2020 Apr 29;9:46. doi: 10.1186/s40249-020-00664-9 (PMC7191747; doi:10.1186/s40249-020-00664-9)
Supplement: Supplementary file 1 — Additional file 1: Table S1. Genotype frequencies of 64 genotyped SNPs in the IL-10, IL18R1, IL1A, IL1B, STAT1, SLC11A1, SP110, IL12B, LTA, TNF, IFNGR1, MBL2, VDR, IL27, CCL2, IL12RB1, IFNGR2 and TLR8 genes among cases and controls and their associations with tuberculosis risk under a codominant genetic model. [file 40249_2020_664_MOESM1_ESM.docx]

Table S1. Genotype frequencies of 64 genotyped SNPs in the *IL10, IL18R1, IL1A, IL1B, STAT1, SLC11A1, SP110, IL12B, LTA, TNF, IFNGR1, MBL2, VDR, IL27, CCL2, IL12RB1, IFNGR2*, and *TLR8* genes among cases and controls and their associations with tuberculosis risk under a codominant genetic model.

| Gene | SNP no. | SNP ID | Genotype | Case（TB） | | Control | | *P* (2 df)^a^ | Logistic Regression | | |
| --- | --- | --- | --- | --- | --- | --- | --- | --- | --- | --- | --- |
|  |  |  |  | No. | Frequency | No. | Frequency |  | OR (95%CI) | *P*^b^ | *P_adjusted_* ^d^ |
| *IL10* | 1 | rs3024496 | T/T | 904 | 90.58% | 455 | 90.10% | 0.9361 | 1.000 (referent) |  |  |
|  |  |  | T/C | 91 | 9.12% | 48 | 9.50% |  | 0.892 (0.606-1.313) | 0.562 | 1.000 |
|  |  |  | C/C | 3 | 0.30% | 2 | 0.40% |  | 0.535 (0.087-3.293) | 0.500 | 1.000 |
|  | 2 | rs1800871 | T/T | 437 | 44.41% | 207 | 41.15% | 0.5266 | 1.000 (referent) |  |  |
|  |  |  | C/T | 422 | 42.89% | 226 | 44.93% |  | 0.874 (0.684-1.117) | 0.283 | 1.000 |
|  |  |  | C/C | 125 | 12.70% | 70 | 13.92% |  | 0.794 (0.556-1.135) | 0.206 | 1.000 |
|  | 3 | rs1800896 | A/A | 829 | 82.82% | 417 | 82.41% | 0.6486 | 1.000 (referent) |  |  |
|  |  |  | G/A | 159 | 15.88% | 85 | 16.80% |  | 0.905 (0.668-1.227) | 0.522 | 1.000 |
|  |  |  | G/G | 13 | 1.30% | 4 | 0.79% |  | 1.234 (0.382-3.988) | 0.726 | 1.000 |
| *IL18R1* | 1 | rs3771167 | T/T | 932 | 93.11% | 471 | 93.08% | 0.6008 | 1.000 (referent) |  |  |
|  |  |  | C/T | 67 | 6.69% | 35 | 6.92% |  | 0.908 (0.578-1.425) | 0.673 | 1.000 |
|  |  |  | C/C | 2 | 0.20% | 0 | 0.00% |  | NA^c^ | NA^c^ | NA^c^ |
|  | 2 | rs1974675 | C/C | 768 | 76.95% | 377 | 74.51% | 0.4062 | 1.000 (referent) |  |  |
|  |  |  | C/T | 208 | 20.84% | 120 | 23.72% |  | 0.791 (0.602-1.039) | 0.092 | 1.000 |
|  |  |  | T/T | 22 | 2.20% | 9 | 1.78% |  | 1.009 (0.445-2.287) | 0.984 | 1.000 |
|  | 3 | rs6758936 | G/G | 737 | 73.77% | 371 | 73.47% | 0.9411 | 1.000 (referent) |  |  |
|  |  |  | G/A | 243 | 24.32% | 125 | 24.75% |  | 0.906 (0.695-1.180) | 0.462 | 1.000 |
|  |  |  | A/A | 19 | 1.90% | 9 | 1.78% |  | 0.863 (0.368-2.021) | 0.734 | 1.000 |
|  | 4 | rs6750020 | G/G | 325 | 32.60% | 139 | 27.58% | 0.0591 | 1.000 (referent) |  |  |
|  |  |  | A/G | 462 | 46.34% | 267 | 52.98% |  | 0.708 (0.544-0.922) | 0.010 | 0.64 |
|  |  |  | A/A | 210 | 21.06% | 98 | 19.44% |  | 0.799 (0.574-1.113) | 0.184 | 1.000 |
|  | 5 | rs1035130 | G/G | 493 | 49.60% | 228 | 45.06% | 0.1718 | 1.000 (referent) |  |  |
|  |  |  | G/A | 408 | 41.05% | 235 | 46.44% |  | 0.799 (0.630-1.014) | 0.064 | 1.000 |
|  |  |  | A/A | 93 | 9.36% | 43 | 8.50% |  | 0.906 (0.595-1.378) | 0.643 | 1.000 |
|  | 6 | rs3771158 | T/T | 834 | 83.40% | 409 | 81.47% | 0.5404 | 1.000 (referent) |  |  |
|  |  |  | C/T | 156 | 15.60% | 89 | 17.73% |  | 0.808 (0.597-1.094) | 0.169 | 1.000 |
|  |  |  | C/C | 10 | 1.00% | 4 | 0.80% |  | 1.040 (0.306-3.537) | 0.950 | 1.000 |
| *IL1A* | 1 | rs17561 | G/G | 808 | 81.21% | 391 | 77.27% | 0.1094 | 1.000 (referent) |  |  |
|  |  |  | G/T | 178 | 17.89% | 108 | 21.34% |  | 0.827 (0.623-1.098) | 0.189 | 1.000 |
|  |  |  | T/T | 9 | 0.90% | 7 | 1.38% |  | 0.588 (0.204-1.698) | 0.326 | 1.000 |
|  | 2 | rs3783526 | A/A | 383 | 38.65% | 226 | 44.66% | 0.0064 | 1.000 (referent) |  |  |
|  |  |  | G/A | 488 | 49.24% | 205 | 40.51% |  | 1.510 (1.181-1.930) | 0.001 | 0.064 |
|  |  |  | G/G | 120 | 12.11% | 75 | 14.82% |  | 1.072 (0.752-1.529) | 0.700 | 1.000 |
| *IL1B* | 1 | rs2853550 | C/C | 789 | 78.90% | 397 | 78.46% | 0.404 | 1.000 (referent) |  |  |
|  |  |  | T/C | 198 | 19.80% | 106 | 20.95% |  | 0.993 (0.750-1.314) | 0.961 | 1.000 |
|  |  |  | T/T | 13 | 1.30% | 3 | 0.59% |  | 2.192 (0.585-8.212) | 0.244 | 1.000 |
|  | 2 | rs1143633 | A/A | 318 | 32.22% | 158 | 31.35% | 0.9139 | 1.000 (referent) |  |  |
|  |  |  | A/G | 497 | 50.35% | 254 | 50.40% |  | 0.957 (0.739-1.238) | 0.738 | 1.000 |
|  |  |  | G/G | 172 | 17.43% | 92 | 18.25% |  | 0.860 (0.616-1.202) | 0.379 | 1.000 |
|  | 3 | rs1143627 | T/T | 260 | 27.03% | 125 | 25.00% | 0.6425 | 1.000 (referent) |  |  |
|  |  |  | C/T | 463 | 48.13% | 253 | 50.60% |  | 0.826 (0.625-1.092) | 0.180 | 1.000 |
|  |  |  | C/C | 239 | 24.84% | 122 | 24.40% |  | 0.822 (0.595-1.137) | 0.237 | 1.000 |
| *STAT1* | 1 | rs2280235 | C/C | 272 | 27.64% | 128 | 25.45% | 0.571 | 1.000 (referent) |  |  |
|  |  |  | C/T | 508 | 51.63% | 263 | 52.29% |  | 0.999 (0.761-1.313) | 0.997 | 1.000 |
|  |  |  | T/T | 204 | 20.73% | 112 | 22.27% |  | 0.973 (0.699-1.354) | 0.871 | 1.000 |
|  | 2 | rs16833155 | C/C | 896 | 89.69% | 453 | 89.53% | 0.7885 | 1.000 (referent) |  |  |
|  |  |  | C/T | 99 | 9.91% | 52 | 10.28% |  | 1.009 (0.693-1.469) | 0.962 | 1.000 |
|  |  |  | T/T | 4 | 0.40% | 1 | 0.20% |  | 3.099 (0.305-31.501) | 0.339 | 1.000 |
|  | 3 | rs13029247 | C/C | 289 | 29.31% | 128 | 25.45% | 0.1559 | 1.000 (referent) |  |  |
|  |  |  | C/T | 483 | 48.99% | 271 | 53.88% |  | 0.822 (0.627-1.077) | 0.155 | 1.000 |
|  |  |  | T/T | 214 | 21.70% | 104 | 20.68% |  | 0.922 (0.662-1.284) | 0.631 | 1.000 |
|  | 4 | rs7576984 | C/C | 693 | 69.51% | 355 | 70.16% | 0.0335 | 1.000 (referent) |  |  |
|  |  |  | C/A | 288 | 28.89% | 133 | 26.28% |  | 1.132 (0.876-1.462) | 0.345 | 1.000 |
|  |  |  | A/A | 16 | 1.60% | 18 | 3.56% |  | 0.479 (0.231-0.992) | 0.048 | 1.000 |
|  | 5 | rs2066802 | T/T | 600 | 60.54% | 312 | 61.90% | 0.006 | 1.000 (referent) |  |  |
|  |  |  | C/T | 365 | 36.83% | 164 | 32.54% |  | 1.172 (0.919-1.494) | 0.201 | 1.000 |
|  |  |  | C/C | 26 | 2.62% | 28 | 5.56% |  | 0.488 (0.272-0.877) | 0.016 | 1.000 |
| *SLC11A1* | 1 | rs2276631 | G/G | 722 | 72.93% | 368 | 72.87% | 0.3192 | 1.000 (referent) |  |  |
|  |  |  | G/A | 240 | 24.24% | 129 | 25.54% |  | 0.905 (0.696-1.178) | 0.458 | 1.000 |
|  |  |  | A/A | 28 | 2.83% | 8 | 1.58% |  | 1.734 (0.756-3.978) | 0.194 | 1.000 |
|  | 2 | rs17221959 | C/C | 738 | 79.01% | 409 | 81.15% | 0.5817 | 1.000 (referent) |  |  |
|  |  |  | C/T | 183 | 19.59% | 90 | 17.86% |  | 1.109 (0.823-1.494) | 0.497 | 1.000 |
|  |  |  | T/T | 13 | 1.39% | 5 | 0.99% |  | 1.369 (0.444-4.223) | 0.585 | 1.000 |
|  | 3 | rs17235409 | G/G | 759 | 76.67% | 373 | 74.01% | 0.5072 | 1.000 (referent) |  |  |
|  |  |  | G/A | 215 | 21.72% | 123 | 24.40% |  | 0.841 (0.641-1.102) | 0.209 | 1.000 |
|  |  |  | A/A | 16 | 1.62% | 8 | 1.59% |  | 0.834 (0.341-2.042) | 0.691 | 1.000 |
| *SP110* | 1 | rs9783992 | T/T | 996 | 99.60% | 504 | 99.60% | 1 | 1.000 (referent) |  |  |
|  |  |  | T/C | 4 | 0.40% | 2 | 0.40% |  | 1.462 (0.246-8.704) | 0.677 | 1.000 |
|  |  |  | C/C | 0 | 0.00% | 0 | 0.00% |  | NA^c^ | NA^c^ | NA^c^ |
|  | 2 | rs10165685 | G/G | 711 | 71.17% | 358 | 70.89% | 0.9726 | 1.000 (referent) |  |  |
|  |  |  | A/G | 263 | 26.33% | 133 | 26.34% |  | 0.957 (0.739-1.239) | 0.738 | 1.000 |
|  |  |  | A/A | 25 | 2.50% | 14 | 2.77% |  | 0.848 (0.420-1.713) | 0.646 | 1.000 |
|  | 3 | rs957683 | T/T | 297 | 30.24% | 158 | 31.23% | 0.2749 | 1.000 (referent) |  |  |
|  |  |  | T/C | 497 | 50.61% | 235 | 46.44% |  | 1.047 (0.805-1.361) | 0.734 | 1.000 |
|  |  |  | C/C | 188 | 19.14% | 113 | 22.33% |  | 0.830 (0.602-1.145) | 0.256 | 1.000 |
|  | 4 | rs41345344 | C/C | 653 | 66.29% | 342 | 68.40% | 0.5347 | 1.000 (referent) |  |  |
|  |  |  | G/C | 300 | 30.46% | 146 | 29.20% |  | 1.082 (0.843-1.390) | 0.535 | 1.000 |
|  |  |  | G/G | 32 | 3.25% | 12 | 2.40% |  | 1.484 (0.734-2.999) | 0.272 | 1.000 |
|  | 5 | rs1365776 | A/A | 779 | 78.13% | 383 | 75.99% | 0.6346 | 1.000 (referent) |  |  |
|  |  |  | A/G | 208 | 20.86% | 115 | 22.82% |  | 0.910 (0.692-1.197) | 0.499 | 1.000 |
|  |  |  | G/G | 10 | 1.00% | 6 | 1.19% |  | 0.689 (0.236-2.008) | 0.494 | 1.000 |
| *IL12B* | 1 | rs1368439 | T/T | 993 | 99.50% | 502 | 99.21% | 0.4801 | 1.000 (referent) |  |  |
|  |  |  | G/T | 5 | 0.50% | 4 | 0.79% |  | 0.700 (0.181-2.706) | 0.605 | 1.000 |
|  |  |  | G/G | 0 | 0.00% | 0 | 0.00% |  | NA^c^ | NA^c^ | NA^c^ |
|  | 2 | rs919766 | A/A | 898 | 89.71% | 454 | 89.72% | 0.6771 | 1.000 (referent) |  |  |
|  |  |  | C/A | 99 | 9.89% | 51 | 10.08% |  | 0.856 (0.587-1.249) | 0.420 | 1.000 |
|  |  |  | C/C | 4 | 0.40% | 1 | 0.20% |  | 1.536 (0.148-15.888) | 0.719 | 1.000 |
|  | 3 | rs3212217 | G/G | 327 | 32.86% | 143 | 28.26% | 0.203 | 1.000 (referent) |  |  |
|  |  |  | G/C | 487 | 48.94% | 266 | 52.57% |  | 0.799 (0.615-1.037) | 0.091 | 1.000 |
|  |  |  | C/C | 181 | 18.19% | 97 | 19.17% |  | 0.860 (0.616-1.201) | 0.377 | 1.000 |
|  | 4 | rs2546892 | G/G | 591 | 62.28% | 334 | 66.14% | 0.3497 | 1.000 (referent) |  |  |
|  |  |  | A/G | 314 | 33.09% | 154 | 30.50% |  | 1.215 (0.947-1.559) | 0.126 | 1.000 |
|  |  |  | A/A | 44 | 4.64% | 17 | 3.37% |  | 1.623 (0.883-2.985) | 0.119 | 1.000 |
| *LTA* | 1 | rs2009658 | C/C | 680 | 68.55% | 373 | 73.72% | 0.115 | 1.000 (referent) |  |  |
|  |  |  | G/C | 284 | 28.63% | 120 | 23.72% |  | 1.333 (1.025-1.733) | 0.032 | 1.000 |
|  |  |  | G/G | 28 | 2.82% | 13 | 2.57% |  | 1.246 (0.614-2.528) | 0.543 | 1.000 |
|  | 2 | rs1800683 | G/G | 335 | 34.11% | 163 | 32.21% | 0.56 | 1.000 (referent) |  |  |
|  |  |  | G/A | 470 | 47.86% | 243 | 48.02% |  | 0.956 (0.739-1.237) | 0.734 | 1.000 |
|  |  |  | A/A | 177 | 18.02% | 100 | 19.76% |  | 0.951 (0.685-1.321) | 0.765 | 1.000 |
|  | 3 | rs2229094 | T/T | 591 | 59.52% | 335 | 66.47% | 0.0329 | 1.000 (referent) |  |  |
|  |  |  | C/T | 349 | 35.15% | 146 | 28.97% |  | 1.388 (1.081-1.782) | 0.010 | 0.64 |
|  |  |  | C/C | 53 | 5.34% | 23 | 4.56% |  | 1.272 (0.743-2.178) | 0.380 | 1.000 |
|  | 4 | rs2229092 | A/A | 954 | 95.40% | 486 | 96.05% | 0.7691 | 1.000 (referent) |  |  |
|  |  |  | C/A | 45 | 4.50% | 19 | 3.75% |  | 1.432 (0.803-2.553) | 0.223 | 1.000 |
|  |  |  | C/C | 1 | 0.10% | 1 | 0.20% |  | 0.534 (0.033-8.579) | 0.658 | 1.000 |
|  | 5 | rs1041981 | C/C | 333 | 33.77% | 163 | 32.21% | 0.5327 | 1.000 (referent) |  |  |
|  |  |  | C/A | 480 | 48.68% | 244 | 48.22% |  | 0.993 (0.768-1.284) | 0.955 | 1.000 |
|  |  |  | A/A | 173 | 17.55% | 99 | 19.57% |  | 0.930 (0.669-1.292) | 0.664 | 1.000 |
| *TNF* | 1 | rs1800629 | G/G | 863 | 86.65% | 452 | 89.86% | 0.1716 | 1.000 (referent) |  |  |
|  |  |  | G/A | 128 | 12.85% | 48 | 9.54% |  | 1.413 (0.976-2.046) | 0.067 | 1.000 |
|  |  |  | A/A | 5 | 0.50% | 3 | 0.60% |  | 0.947 (0.210-4.273) | 0.943 | 1.000 |
|  | 2 | rs3093662 | A/A | 899 | 90.53% | 467 | 92.66% | 0.4052 | 1.000 (referent) |  |  |
|  |  |  | G/A | 92 | 9.26% | 36 | 7.14% |  | 1.213 (0.790-1.862) | 0.378 | 1.000 |
|  |  |  | G/G | 2 | 0.20% | 1 | 0.20% |  | 0.918 (0.076-11.078) | 0.946 | 1.000 |
| *IFNGR1* | 1 | rs1887415 | T/T | 938 | 93.80% | 474 | 94.23% | 0.7139 | 1.000 (referent) |  |  |
|  |  |  | T/C | 62 | 6.20% | 29 | 5.77% |  | 1.178 (0.725-1.914) | 0.509 | 1.000 |
|  |  |  | C/C | 0 | 0.00% | 0 | 0.00% |  | NA^c^ | NA^c^ | NA^c^ |
|  | 2 | rs2234711 | C/C | 314 | 31.72% | 145 | 28.66% | 0.1484 | 1.000 (referent) |  |  |
|  |  |  | T/C | 465 | 46.97% | 264 | 52.17% |  | 0.755 (0.580-0.982) | 0.036 | 1.000 |
|  |  |  | T/T | 211 | 21.31% | 97 | 19.17% |  | 0.944 (0.678-1.314) | 0.733 | 1.000 |
| *MBL2* | 1 | rs2099902 | T/T | 552 | 55.76% | 323 | 63.83% | 0.0025 | 1.000 (referent) |  |  |
|  |  |  | C/T | 372 | 37.58% | 166 | 32.81% |  | 2.364 (1.317-4.244) | 0.004 | 0.256 |
|  |  |  | C/C | 66 | 6.67% | 17 | 3.36% |  | 1.459 (1.143-1.863) | 0.002 | 0.128 |
|  | 2 | **rs930507** | C/C | 549 | 57.31% | 330 | 65.35% | 0.0149 | 1.000 (referent) |  |  |
|  |  |  | C/G | 363 | 37.89% | 157 | 31.09% |  | 1.556 (1.215-1.992) | 4.218E-4 | 0.027 |
|  |  |  | G/G | 46 | 4.80% | 18 | 3.56% |  | 1.672 (0.921-3.038) | 0.091 | 1.000 |
|  | 3 | rs10824793 | A/A | 434 | 43.44% | 259 | 51.19% | 0.0171 | 1.000 (referent) |  |  |
|  |  |  | G/A | 459 | 45.95% | 206 | 40.71% |  | 1.466 (1.153-1.863) | 0.002 | 0.128 |
|  |  |  | G/G | 106 | 10.61% | 41 | 8.10% |  | 1.890 (1.245-2.870) | 0.003 | 0.192 |
|  | 4 | rs7916582 | T/T | 762 | 76.28% | 398 | 79.28% | 0.3794 | 1.000 (referent) |  |  |
|  |  |  | T/C | 223 | 22.32% | 97 | 19.32% |  | 1.338 (1.006-1.779) | 0.045 | 1.000 |
|  |  |  | C/C | 14 | 1.40% | 7 | 1.39% |  | 1.134 (0.424-3.034) | 0.803 | 1.000 |
| *VDR* | 1 | rs2239184 | C/C | 533 | 53.41% | 264 | 52.17% | 0.9077 | 1.000 (referent) |  |  |
|  |  |  | C/T | 390 | 39.08% | 202 | 39.92% |  | 0.981 (0.773-1.247) | 0.878 | 1.000 |
|  |  |  | T/T | 75 | 7.52% | 40 | 7.91% |  | 0.963 (0.622-1.491) | 0.866 | 1.000 |
|  | 2 | rs2248098 | T/T | 518 | 51.96% | 259 | 51.29% | 0.5545 | 1.000 (referent) |  |  |
|  |  |  | T/C | 398 | 39.92% | 197 | 39.01% |  | 1.020 (0.802-1.297) | 0.874 | 1.000 |
|  |  |  | C/C | 81 | 8.12% | 49 | 9.70% |  | 0.853 (0.566-1.285) | 0.447 | 1.000 |
|  | 3 | rs1540339 | A/A | 468 | 46.94% | 252 | 49.80% | 0.2301 | 1.000 (referent) |  |  |
|  |  |  | G/A | 428 | 42.93% | 216 | 42.69% |  | 1.111 (0.876-1.409) | 0.386 | 1.000 |
|  |  |  | G/G | 101 | 10.13% | 38 | 7.51% |  | 1.483 (0.969-2.271) | 0.070 | 1.000 |
|  | 4 | rs10783219 | A/A | 243 | 26.47% | 166 | 33.00% | 0.0239 | 1.000 (referent) |  |  |
|  |  |  | T/A | 497 | 54.14% | 238 | 47.32% |  | 1.407 (1.079-1.835) | 0.012 | 0.768 |
|  |  |  | T/T | 178 | 19.39% | 99 | 19.68% |  | 1.207 (0.863-1.687) | 0.272 | 1.000 |
|  | 5 | rs7139166 | C/C | 947 | 94.70% | 478 | 94.47% | 0.8153 | 1.000 (referent) |  |  |
|  |  |  | C/G | 53 | 5.30% | 28 | 5.53% |  | 0.887 (0.537-1.466) | 0.641 | 1.000 |
|  |  |  | G/G | 0 | 0.00% | 0 | 0.00% |  | NA^c^ | NA^c^ | NA^c^ |
| *IL27* | 1 | rs181206 | T/T | 743 | 75.28% | 375 | 74.26% | 0.8264 | 1.000 (referent) |  |  |
|  |  |  | T/C | 233 | 23.61% | 123 | 24.36% |  | 0.915 (0.701-1.195) | 0.515 | 1.000 |
|  |  |  | C/C | 11 | 1.11% | 7 | 1.39% |  | 0.809 (0.295-2.216) | 0.680 | 1.000 |
| *CCL2* | 1 | rs4586 | C/C | 391 | 39.57% | 175 | 34.65% | 0.1794 | 1.000 (referent) |  |  |
|  |  |  | T/C | 443 | 44.84% | 244 | 48.32% |  | 0.831 (0.646-1.068) | 0.148 | 1.000 |
|  |  |  | T/T | 154 | 15.59% | 86 | 17.03% |  | 0.816 (0.583-1.143) | 0.237 | 1.000 |
| *IL12RB1* | 1 | rs2305740 | A/A | 786 | 78.60% | 378 | 74.85% | 0.0198 | 1.000 (referent) |  |  |
|  |  |  | A/G | 208 | 20.80% | 117 | 23.17% |  | 0.828 (0.630-1.089) | 0.178 | 1.000 |
|  |  |  | G/G | 6 | 0.60% | 10 | 1.98% |  | 0.288 (0.101-0.826) | 0.021 | 1.000 |
|  | 2 | rs401502 | C/C | 425 | 42.59% | 205 | 40.51% | 0.3192 | 1.000 (referent) |  |  |
|  |  |  | G/C | 459 | 45.99% | 229 | 45.26% |  | 0.913 (0.715-1.165) | 0.463 | 1.000 |
|  |  |  | G/G | 114 | 11.42% | 72 | 14.23% |  | 0.784 (0.547-1.123) | 0.184 | 1.000 |
|  | 3 | rs375947 | A/A | 426 | 42.77% | 205 | 40.67% | 0.2013 | 1.000 (referent) |  |  |
|  |  |  | A/G | 458 | 45.98% | 227 | 45.04% |  | 0.918 (0.719-1.172) | 0.492 | 1.000 |
|  |  |  | G/G | 112 | 11.24% | 72 | 14.29% |  | 0.765 (0.534-1.097) | 0.146 | 1.000 |
|  | 4 | rs17852635 | G/G | 433 | 43.87% | 205 | 40.51% | 0.079 | 1.000 (referent) |  |  |
|  |  |  | G/A | 452 | 45.80% | 230 | 45.45% |  | 0.868 (0.680-1.108) | 0.257 | 1.000 |
|  |  |  | A/A | 102 | 10.33% | 71 | 14.03% |  | 0.689 (0.477-0.995) | 0.047 | 1.000 |
|  | 5 | rs11575934 | A/A | 426 | 43.16% | 196 | 39.04% | 0.1176 | 1.000 (referent) |  |  |
|  |  |  | A/G | 452 | 45.80% | 235 | 46.81% |  | 0.827 (0.647-1.057) | 0.130 | 1.000 |
|  |  |  | G/G | 109 | 11.04% | 71 | 14.14% |  | 0.716 (0.497-1.031) | 0.073 | 1.000 |
| *IFNGR2* | 1 | rs1059293 | T/T | 783 | 78.77% | 397 | 78.46% | 0.3319 | 1.000 (referent) |  |  |
|  |  |  | T/C | 196 | 19.72% | 96 | 18.97% |  | 1.009 (0.757-1.345) | 0.952 | 1.000 |
|  |  |  | C/C | 15 | 1.51% | 13 | 2.57% |  | 0.650 (0.291-1.448) | 0.292 | 1.000 |
| *TLR8* | 1 | rs3764880 | G/G | 787 | 79.18% | 397 | 78.61% | 0.6307 | 1.000 (referent) |  |  |
|  |  |  | G/A | 106 | 10.66% | 61 | 12.08% |  | 1.021 (0.690-1.510) | 0.919 | 1.000 |
|  |  |  | A/A | 101 | 10.16% | 47 | 9.31% |  | 0.927 (0.626-1.372) | 0.705 | 1.000 |
|  | 2 | rs5744068 | C/C | 973 | 97.40% | 493 | 97.43% | 0.6079 | 1.000 (referent) |  |  |
|  |  |  | C/T | 14 | 1.40% | 9 | 1.78% |  | 0.828 (0.336-2.044) | 0.683 | 1.000 |
|  |  |  | T/T | 12 | 1.20% | 4 | 0.79% |  | 1.276 (0.383-4.250) | 0.691 | 1.000 |
|  | 3 | rs2159377 | T/T | 717 | 72.35% | 359 | 71.09% | 0.6886 | 1.000 (referent) |  |  |
|  |  |  | C/T | 141 | 14.23% | 80 | 15.84% |  | 0.985 (0.685-1.417) | 0.934 | 1.000 |
|  |  |  | C/C | 133 | 13.42% | 66 | 13.07% |  | 0.952 (0.676-1.340) | 0.776 | 1.000 |
|  | 4 | rs5744080 | T/T | 750 | 75.15% | 370 | 73.41% | 0.6444 | 1.000 (referent) |  |  |
|  |  |  | C/T | 128 | 12.83% | 72 | 14.29% |  | 0.977 (0.673-1.418) | 0.902 | 1.000 |
|  |  |  | C/C | 120 | 12.02% | 62 | 12.30% |  | 0.858 (0.603-1.218) | 0.391 | 1.000 |
|  | 5 | rs2407992 | C/C | 753 | 75.98% | 372 | 74.10% | 0.6905 | 1.000 (referent) |  |  |
|  |  |  | G/C | 125 | 12.61% | 69 | 13.75% |  | 1.015 (0.696-1.481) | 0.937 | 1.000 |
|  |  |  | G/G | 113 | 11.40% | 61 | 12.15% |  | 0.801 (0.561-1.145) | 0.223 | 1.000 |
|  | 6 | rs3747414 | A/A | 748 | 75.86% | 371 | 73.32% | 0.6069 | 1.000 (referent) |  |  |
|  |  |  | C/A | 126 | 12.78% | 72 | 14.23% |  | 0.973 (0.671-1.412) | 0.886 | 1.000 |
|  |  |  | C/C | 112 | 11.36% | 63 | 12.45% |  | 0.804 (0.565-1.145) | 0.226 | 1.000 |
|  | 7 | rs5744088 | G/G | 949 | 96.44% | 487 | 96.25% | 0.9646 | 1.000 (referent) |  |  |
|  |  |  | G/C | 22 | 2.24% | 13 | 2.57% |  | 0.957 (0.455-2.012) | 0.907 | 1.000 |
|  |  |  | C/C | 13 | 1.32% | 6 | 1.19% |  | 1.045 (0.364-3.000) | 0.934 | 1.000 |

a. Global *P* values [2 degrees of freedom (df)]: genotype frequencies in tuberculosis and control group were compared using a χ^2^ test with two df.

b. *P* values from unconditional logistic regression analyses, adjusted for age and gender.

c. NA, not available because of the rarity of genotype.

d. *P_adjusted_*, P value with Bonferroni correction, *P_adjusted_* value less than 0.05 was considered to be significant. NS, no significance.
