# Supplementary material for: Mannose-binding lectin 2 gene polymorphisms and their association with tuberculosis in a Chinese population
Source: Infect Dis Poverty. 2020 Apr 29;9:46. doi: 10.1186/s40249-020-00664-9 (PMC7191747; doi:10.1186/s40249-020-00664-9)
Supplement: Supplementary file 2 — Additional file 2: Table S2.. Association analysis of 64 SNPs in the IL-10, IL18R1, IL1A, IL1B, STAT1, SLC11A1, SP110, IL12B, LTA, TNF, IFNGR1, MBL2, VDR, IL27, CCL2, IL12RB1, IFNGR2 and TLR8 genes under a dominant and recessive genetic model. [file 40249_2020_664_MOESM2_ESM.docx]

**Table S2. Association analysis of 64 SNPs in the *IL10, IL18R1, IL1A, IL1B, STAT1, SLC11A1, SP110, IL12B, LTA, TNF, IFNGR1, MBL2, VDR, IL27, CCL2, IL12RB1, IFNGR2,* and *TLR8* genes under a dominant and recessive genetic model.**

| Gene | SNP no. | SNP ID | Genetic model | Case | Control | Logistic Regression | | |
| --- | --- | --- | --- | --- | --- | --- | --- | --- |
|  |  |  |  |  |  | OR (95%CI) | *P*^b^ | *Pc*^d^ |
| IL10 | 1 | rs3024496 | (TC+CC) vs. TT | 94/904 | 50/455 | 0.875 (0.599-1.280) | 0.492 | 1.000 |
|  |  |  | CC vs. (TT+TC) | 3/995 | 2/503 | 0.541 (0.088-3.329) | 0.508 | 1.000 |
|  | 2 | rs1800871 | (CT+CC) vs. TT | 547/437 | 296/207 | 0.855 (0.679-1.077) | 0.184 | 1.000 |
|  |  |  | CC vs. (TT+CT) | 125/859 | 70/433 | 0.851 (0.609-1.188) | 0.342 | 1.000 |
|  | 3 | rs1800896 | (GA+GG) vs. AA | 172/829 | 89/417 | 0.921 (0.685-1.240) | 0.590 | 1.000 |
|  |  |  | GG vs. (AA+GA) | 13/988 | 4/502 | 1.254 (0.388-4.051) | 0.705 | 1.000 |
| IL18R1 | 1 | rs3771167 | (CT+CC) vs. TT | 69/932 | 35/471 | 0.924 (0.589-1.447) | 0.728 | 1.000 |
|  |  |  | CC vs. (TT+CT) | 2/999 | 0/506 | NA^c^ | NA^c^ | NA^c^ |
|  | 2 | rs1974675 | (CT+TT) vs. CC | 230/768 | 129/377 | 0.807 (0.620-1.051) | 0.112 | 1.000 |
|  |  |  | TT vs. (CC+CT) | 22/976 | 9/497 | 1.064 (0.471-2.406) | 0.881 | 1.000 |
|  | 3 | rs6758936 | (GA+AA) vs. GG | 262/737 | 134/371 | 0.903 (0.698-1.168) | 0.436 | 1.000 |
|  |  |  | AA vs. (GG+GA) | 19/980 | 9/496 | 0.885 (0.379-2.066) | 0.777 | 1.000 |
|  | 4 | rs6750020 | (AG+AA) vs. GG | 672/325 | 365/139 | 0.733 (0.571-0.941) | 0.015 | 0.96 |
|  |  |  | AA vs. (GG+AG) | 210/787 | 98/406 | 0.991 (0.745-1.318) | 0.950 | 1.000 |
|  | 5 | rs1035130 | (GA+AA) vs. GG | 501/493 | 278/228 | 0.816 (0.650-1.024) | 0.080 | 1.000 |
|  |  |  | AA vs. (GG+GA) | 93/901 | 43/463 | 1.009 (0.674-1.509) | 0.966 | 1.000 |
|  | 6 | rs3771158 | (CT+CC) vs. TT | 166/834 | 93/409 | 0.819 (0.609-1.102) | 0.186 | 1.000 |
|  |  |  | CC vs. (TT+CT) | 10/990 | 4/498 | 1.078 (0.317-3.659) | 0.905 | 1.000 |
| IL1A | 1 | rs17561 | (GT+TT) vs. GG | 187/808 | 115/391 | 0.812 (0.616-1.071) | 0.141 | 1.000 |
|  |  |  | TT vs. (GG+GT) | 9/986 | 7/499 | 0.611 (0.212-1.760) | 0.361 | 1.000 |
|  | 2 | rs3783526 | (GA+GG) vs. AA | 608/383 | 280/226 | 1.396 (1.109-1.758) | 0.005 | 0.32 |
|  |  |  | GG vs. (AA+GA) | 120/871 | 75/431 | 0.865 (0.621-1.205) | 0.392 | 1.000 |
| IL1B | 1 | rs2853550 | (TC+TT) vs. CC | 211/789 | 109/397 | 1.026 (0.779-1.352) | 0.855 | 1.000 |
|  |  |  | TT vs. (CC+TC) | 13/987 | 3/503 | 2.195 (0.587-8.214) | 0.243 | 1.000 |
|  | 2 | rs1143633 | (AG+GG) vs. AA | 669/318 | 346/158 | 0.931 (0.729-1.188) | 0.564 | 1.000 |
|  |  |  | GG vs. (AA+AG) | 172/815 | 92/412 | 0.884 (0.658-1.187) | 0.412 | 1.000 |
|  | 3 | rs1143627 | (CT+CC) vs. TT | 702/260 | 375/125 | 0.825 (0.634-1.073) | 0.151 | 1.000 |
|  |  |  | CC vs. (TT+CT) | 239/723 | 122/378 | 0.932 (0.714-1.217) | 0.604 | 1.000 |
| STAT1 | 1 | rs2280235 | (CT+TT) vs. CC | 712/272 | 375/128 | 0.992 (0.765-1.285) | 0.949 | 1.000 |
|  |  |  | TT vs. (CC+CT) | 204/780 | 112/391 | 0.973 (0.739-1.282) | 0.847 | 1.000 |
|  | 2 | rs16833155 | (CT+TT) vs. CC | 103/896 | 53/453 | 1.042 (0.718-1.510) | 0.830 | 1.000 |
|  |  |  | TT vs. (CC+CT) | 4/995 | 1/505 | 3.096 (0.305-31.461) | 0.339 | 1.000 |
|  | 3 | rs13029247 | (CT+TT) vs. CC | 697/289 | 375/128 | 0.850 (0.657-1.099) | 0.215 | 1.000 |
|  |  |  | TT vs. (CC+CT) | 214/772 | 104/399 | 1.048 (0.794-1.385) | 0.739 | 1.000 |
|  | 4 | rs7576984 | (CA+AA) vs. CC | 304/693 | 151/355 | 1.054 (0.823-1.349) | 0.677 | 1.000 |
|  |  |  | AA vs. (CC+CA) | 16/981 | 18/488 | 0.463 (0.224-0.955) | 0.037 | 1.000 |
|  | 5 | rs2066802 | (CT+CC) vs. TT | 391/600 | 192/312 | 1.071 (0.848-1.353) | 0.562 | 1.000 |
|  |  |  | CC vs. (TT+CT) | 26/965 | 28/476 | 0.461 (0.258-0.823) | 0.009 | 0.576 |
| SLC11A1 | 1 | rs2276631 | (GA+AA) vs. GG | 268/722 | 137/368 | 0.954 (0.738-1.231) | 0.716 | 1.000 |
|  |  |  | AA vs. (GG+GA) | 28/962 | 8/497 | 1.778 (0.777-4.068) | 0.173 | 1.000 |
|  | 2 | rs17221959 | (CT+TT) vs. CC | 196/738 | 95/409 | 1.122 (0.838-1.501) | 0.439 | 1.000 |
|  |  |  | TT vs. (CC+CT) | 13/921 | 5/499 | 1.343 (0.436-4.138) | 0.608 | 1.000 |
|  | 3 | rs17235409 | (GA+GG) vs. AA | 231/759 | 131/373 | 0.840 (0.646-1.094) | 0.196 | 1.000 |
|  |  |  | GG vs. (AA+GA) | 16/974 | 8/496 | 0.867 (0.355-2.118) | 0.754 | 1.000 |
| SP110 | 1 | rs9783992 | (TC+CC) vs. TT | 4/996 | 2/504 | 1.462 (0.246-8.704) | 0.677 | 1.000 |
|  |  |  | CC vs. (TT+TC) | 0/1000 | 0/506 | NA^c^ | NA^c^ | NA^c^ |
|  | 2 | rs10165685 | (AG+AA) vs. GG | 288/711 | 147/358 | 0.946 (0.738-1.214) | 0.665 | 1.000 |
|  |  |  | AA vs. (GG+AG) | 25/974 | 14/491 | 0.858 (0.426-1.728) | 0.669 | 1.000 |
|  | 3 | rs957683 | (TC+CC) vs. TT | 685/297 | 348/158 | 0.976 (0.763-1.249) | 0.846 | 1.000 |
|  |  |  | CC vs. (TT+TC) | 188/794 | 113/393 | 0.807 (0.611-1.067) | 0.133 | 1.000 |
|  | 4 | rs41345344 | (GC+GG) vs. CC | 332/653 | 158/342 | 1.113 (0.873-1.418) | 0.388 | 1.000 |
|  |  |  | GG vs. (CC+GC) | 32/953 | 12/488 | 1.448 (0.719-2.914) | 0.300 | 1.000 |
|  | 5 | rs1365776 | (AG+GG) vs. AA | 218/779 | 121/383 | 0.898 (0.686-1.175) | 0.432 | 1.000 |
|  |  |  | GG vs. (AA+AG) | 10/987 | 6/498 | 0.703 (0.242-2.047) | 0.518 | 1.000 |
| IL12B | 1 | rs1368439 | (GT+GG) vs. TT | 5/993 | 4/502 | 0.700 (0.181-2.706) | 0.605 | 1.000 |
|  |  |  | GG vs. (TT+GT) | 0/998 | 0/506 | NA^c^ | NA^c^ | NA^c^ |
|  | 2 | rs919766 | (CA+CC) vs. AA | 103/898 | 52/454 | 0.869 (0.598-1.262) | 0.462 | 1.000 |
|  |  |  | CC vs. (AA+CA) | 4/997 | 1/505 | 1.560 (0.151-16.113) | 0.709 | 1.000 |
|  | 3 | rs3212217 | (GC+CC) vs. GG | 668/327 | 363/143 | 0.815 (0.636-1.044) | 0.105 | 1.000 |
|  |  |  | CC vs. (GG+GC) | 181/814 | 97/409 | 0.990 (0.741-1.323) | 0.946 | 1.000 |
|  | 4 | rs2546892 | (AG+AA) vs. GG | 358/591 | 171/334 | 1.255 (0.987-1.596) | 0.064 | 1.000 |
|  |  |  | AA vs. (GG+AG) | 44/905 | 17/488 | 1.521 (0.832-2.781) | 0.173 | 1.000 |
| LTA | 1 | rs2009658 | (GC+GG) vs. CC | 312/680 | 133/373 | 1.324 (1.028-1.706) | 0.030 | 1.000 |
|  |  |  | GG vs. (CC+GC) | 28/964 | 13/493 | 1.153 (0.570-2.332) | 0.691 | 1.000 |
|  | 2 | rs1800683 | (GA+AA) vs. GG | 647/335 | 343/163 | 0.955 (0.749-1.217) | 0.709 | 1.000 |
|  |  |  | AA vs. (GG+GA) | 177/805 | 100/406 | 0.977 (0.731-1.306) | 0.873 | 1.000 |
|  | 3 | rs2229094 | (CT+CC) vs. TT | 402/591 | 169/335 | 1.372 (1.081-1.740) | 0.009 | 0.576 |
|  |  |  | CC vs. (TT+CT) | 53/940 | 23/481 | 1.140 (0.670-1.940) | 0.628 | 1.000 |
|  | 4 | rs2229092 | (CA+CC) vs. AA | 46/954 | 20/486 | 1.381 (0.784-2.432) | 0.264 | 1.000 |
|  |  |  | CC vs. (AA+CA) | 1/999 | 1/505 | 0.524 (0.033-8.423) | 0.649 | 1.000 |
|  | 5 | rs1041981 | (CA+AA) vs. CC | 653/333 | 343/163 | 0.975 (0.765-1.242) | 0.836 | 1.000 |
|  |  |  | AA vs. (CC+CA) | 173/813 | 99/407 | 0.934 (0.698-1.250) | 0.644 | 1.000 |
| TNF | 1 | rs1800629 | (GA+AA) vs. GG | 133/863 | 51/452 | 1.386 (0.966-1.989) | 0.076 | 1.000 |
|  |  |  | AA vs. (GG+GA) | 5/991 | 3/500 | 0.911 (0.202-4.108) | 0.903 | 1.000 |
|  | 2 | rs3093662 | (GA+GG) vs. AA | 94/899 | 37/467 | 1.204 (0.788-1.839) | 0.390 | 1.000 |
|  |  |  | GG vs. (AA+GA) | 2/991 | 1/503 | 0.903 (0.075-10.895) | 0.936 | 1.000 |
| IFNGR1 | 1 | rs1887415 | (TC+CC) vs. TT | 62/938 | 29/474 | 1.178 (0.725-1.914) | 0.509 | 1.000 |
|  |  |  | CC vs. (TT+TC) | 0/1000 | 0/503 | NA^c^ | NA^c^ | NA^c^ |
|  | 2 | rs2234711 | (TC+TT) vs. CC | 676/314 | 361/145 | 0.805 (0.627-1.033) | 0.088 | 1.000 |
|  |  |  | TT vs. (CC+TC) | 211/779 | 97/409 | 1.124 (0.845-1.496) | 0.422 | 1.000 |
| MBL2 | 1 | rs2099902 | (CT+CC) vs. TT | 438/552 | 183/323 | 1.544 (1.220-1.954) | 3.023E-3 | **0.020** |
|  |  |  | CC vs. (TT+CT) | 66/924 | 17/489 | 2.055 (1.154-3.659) | 0.014 | 0.896 |
|  | 2 | rs930507 | (CG+GG) vs. CC | 409/549 | 175/330 | 1.568 (1.235-1.990) | 2.211E-4 | **0.027** |
|  |  |  | GG vs. (CC+CG) | 46/912 | 18/487 | 1.425 (0.790-2.568) | 0.239 | 1.000 |
|  | 3 | rs10824793 | (GA+GG) vs. AA | 565/434 | 247/259 | 1.533 (1.219-1.927) | 2.544E-4 | **0.017** |
|  |  |  | GG vs. (AA+GA) | 106/893 | 41/465 | 1.571 (1.052-2.345) | 0.027 | 1.000 |
|  | 4 | rs7916582 | (TC+CC) vs. TT | 237/762 | 104/398 | 1.324 (1.003-1.748) | 0.047 | 1.000 |
|  |  |  | CC vs. (TT+TC) | 14/985 | 7/495 | 1.065 (0.399-2.841) | 0.900 | 1.000 |
|  | 5 | rs10875695 |  |  |  |  |  |  |
|  |  |  |  |  |  |  |  |  |
| VDR | 1 | rs2239184 | (CT+TT) vs. CC | 465/533 | 242/264 | 0.978 (0.779-1.229) | 0.851 | 1.000 |
|  |  |  | TT vs. (CC+CT) | 75/923 | 40/466 | 0.971 (0.635-1.484) | 0.891 | 1.000 |
|  | 2 | rs2248098 | (TC+CC) vs. TT | 479/518 | 246/259 | 0.987 (0.786-1.238) | 0.908 | 1.000 |
|  |  |  | CC vs. (TT+TC) | 81/916 | 49/456 | 0.846 (0.569-1.258) | 0.408 | 1.000 |
|  | 3 | rs1540339 | (GA+GG) vs. AA | 529/468 | 254/252 | 1.166 (0.930-1.464) | 0.183 | 1.000 |
|  |  |  | GG vs. (AA+GA) | 101/896 | 38/468 | 1.412 (0.936-2.129) | 0.100 | 1.000 |
|  | 4 | rs10783219 | (TA+TT) vs. AA | 675/243 | 337/166 | 1.349 (1.049-1.734) | 0.020 | 1.000 |
|  |  |  | TT vs. (AA+TA) | 178/740 | 99/404 | 0.973 (0.727-1.302) | 0.854 | 1.000 |
|  | 5 | rs7139166 | (CG+GG) vs. CC | 53/947 | 28/478 | 0.887 (0.537-1.466) | 0.641 | 1.000 |
|  |  |  | GG vs. (CC+CG) | 0/1000 | 0/506 | 0.827 (0.302-2.259) | 0.710 | 1.000 |
| IL27 | 1 | rs181206 | (TC+CC) vs. TT | 244/743 | 130/375 | 0.909 (0.700-1.181) | 0.477 | 1.000 |
|  |  |  | CC vs. (TT+TC) | 11/976 | 7/498 | NA^c^ | NA^c^ | NA^c^ |
| CCL2 | 1 | rs4586 | (TC+TT) vs. CC | 597/391 | 330/175 | 0.827 (0.653-1.047) | 0.115 | 1.000 |
|  |  |  | TT vs. (CC+TC) | 154/834 | 86/419 | 0.905 (0.667-1.228) | 0.523 | 1.000 |
| IL12RB1 | 1 | rs2305740 | (AG+GG) vs. AA | 214/786 | 127/378 | 0.784 (0.601-1.024) | 0.075 | 1.000 |
|  |  |  | GG vs. (AA+AG) | 6/994 | 10/495 | 0.301 (0.105-0.861) | 0.025 | 1.000 |
|  | 2 | rs401502 | (GC+GG) vs. CC | 573/425 | 301/205 | 0.883 (0.701-1.111) | 0.288 | 1.000 |
|  |  |  | GG vs. (CC+GC) | 114/884 | 72/434 | 0.822 (0.588-1.150) | 0.252 | 1.000 |
|  | 3 | rs375947 | (AG+GG) vs. AA | 570/426 | 299/205 | 0.882 (0.700-1.111) | 0.286 | 1.000 |
|  |  |  | GG vs. (AA+AG) | 112/884 | 72/432 | 0.800 (0.571-1.121) | 0.194 | 1.000 |
|  | 4 | rs17852635 | (GA+AA) vs. GG | 554/433 | 301/205 | 0.827 (0.656-1.042) | 0.107 | 1.000 |
|  |  |  | AA vs. (GG+GA) | 102/885 | 71/435 | 0.741 (0.525-1.046) | 0.088 | 1.000 |
|  | 5 | rs11575934 | (AG+GG) vs. AA | 561/426 | 306/196 | 0.802 (0.636-1.012) | 0.063 | 1.000 |
|  |  |  | GG vs. (AA+AG) | 109/878 | 71/431 | 0.792 (0.564-1.112) | 0.178 | 1.000 |
| IFNGR2 | 1 | rs1059293 | (TC+CC) vs. TT | 211/783 | 109/397 | 0.968 (0.735-1.275) | 0.817 | 1.000 |
|  |  |  | CC vs. (TT+TC) | 15/979 | 13/493 | 0.648 (0.291-1.443) | 0.288 | 1.000 |
| TLR8 | 1 | rs3764880 | (GA+AA) vs. GG | 207/787 | 108/397 | 0.973 (0.732-1.293) | 0.850 | 1.000 |
|  |  |  | AA vs. (GG+GA) | 101/893 | 47/458 | 0.926 (0.626-1.371) | 0.701 | 1.000 |
|  | 2 | rs5744068 | (CT+TT) vs. CC | 26/973 | 13/493 | 0.975 (0.476-1.994) | 0.944 | 1.000 |
|  |  |  | TT vs. (CC+CT) | 12/987 | 4/502 | 1.277 (0.383-4.251) | 0.691 | 1.000 |
|  | 3 | rs2159377 | (CT+CC) vs. TT | 274/717 | 146/359 | 0.967 (0.746-1.254) | 0.800 | 1.000 |
|  |  |  | CC vs. (TT+CT) | 133/858 | 66/439 | 0.953 (0.678-1.339) | 0.781 | 1.000 |
|  | 4 | rs5744080 | (CT+CC) vs. TT | 248/750 | 134/370 | 0.912 (0.699-1.189) | 0.495 | 1.000 |
|  |  |  | CC vs. (TT+CT) | 120/878 | 62/442 | 0.859 (0.605-1.219) | 0.395 | 1.000 |
|  | 5 | rs2407992 | (GC+GG) vs. CC | 238/753 | 130/372 | 0.896 (0.685-1.173) | 0.426 | 1.000 |
|  |  |  | GG vs. (CC+GC) | 113/878 | 61/441 | 0.800 (0.561-1.142) | 0.220 | 1.000 |
|  | 6 | rs3747414 | (CA+CC) vs. AA | 238/748 | 135/371 | 0.880 (0.674-1.149) | 0.349 | 1.000 |
|  |  |  | CC vs. (AA+CA) | 112/874 | 63/443 | 0.806 (0.567-1.146) | 0.229 | 1.000 |
|  | 7 | rs5744088 | (GC+CC) vs. GG | 35/949 | 19/487 | 0.986 (0.537-1.809) | 0.962 | 1.000 |
|  |  |  | CC vs. (GG+GC) | 13/971 | 6/500 | 1.045 (0.364-3.000) | 0.934 | 1.000 |

a. Global *P* values [2 degrees of freedom (df)]: genotype frequencies in tuberculosis and control group were compared using a χ^2^ test with two df.

b. *P* values from unconditional logistic regression analyses, adjusted for age and gender.

c. NA, not available because of the rarity of genotype.

d. *P*c, *P* value with Bonferroni correction, *P*c value less than 0.05 was considered to be significant.
