# Supplementary material for: Mannose-binding lectin 2 gene polymorphisms and their association with tuberculosis in a Chinese population
Source: Infect Dis Poverty. 2020 Apr 29;9:46. doi: 10.1186/s40249-020-00664-9 (PMC7191747; doi:10.1186/s40249-020-00664-9)
Supplement: Supplementary file 3 — Additional file 3: Table S3. Association analysis of MBL2 SNPs between TB subgroups and healthy controls under a codominant genetic model. [file 40249_2020_664_MOESM3_ESM.docx]

**Table S3 Association analysis of *MBL2* SNPs between TB subgroups and healthy controls under a codominant genetic model.**

| **SNP** | **Genotype** | **EPTB** | | | **PTB** | | | **PTB+EPTB** | | | **TPTB** | | |
| --- | --- | --- | --- | --- | --- | --- | --- | --- | --- | --- | --- | --- | --- |
|  |  | ***P* ^a^** | ***P_adjusted_*^b^** | **OR [95% CI] ^c^** | ***P*** | ***P_adjusted_*** | **OR [95% CI]** | ***P*** | ***P_adjusted_*** | **OR [95% CI]** | ***P*** | ***P_adjusted_*** | **OR [95% CI]** |
| rs2099902 | C/T | 0.098 | 1.000 | 1.430(0.936-2.185) | 0.081 | 1.000 | 1.305(0.968-1.758) | 0.144 | 1.000 | 1.389(0.894-2.159) | 0.011 | 0.704 | 1.406(1.082-1.829) |
|  | C/C | 0.037 | 1.000 | 2.461(1.054-5.746) | 0.021 | 1.000 | 2.244(1.131-4.455) | 0.925 | 1.000 | 1.052(0.368-3.007) | 0.005 | 0.320 | 2.408(1.302-4.455) |
| rs930507 | C/G | 0.007 | 0.448 | 1.788(1.169-2.736) | 0.007 | 0.448 | 1.513(1.120-2.044) | 0.089 | 1.000 | 1.469(0.943-2.287) | 0.003 | 0.192 | 1.505(1.153-1.964) |
|  | G/G | 0.448 | 1.000 | 0.603(0.163-2.232) | 0.176 | 1.000 | 1.626(0.805-3.285) | 0.324 | 1.000 | 1.672(0.602-4.644) | 0.105 | 1.000 | 1.674(0.898-3.120) |
| rs10824793 | G/A | 0.103 | 1.000 | 1.413(0.933-2.140) | 0.010 | 0.640 | 1.478(1.099-1.988) | 0.015 | 0.960 | 1.736(1.112-2.710) | 0.004 | 0.256 | 1.463(1.129-1.895) |
|  | G/G | 0.274 | 1.000 | 1.505(0.724-3.128) | 0.006 | 0.384 | 2.003(1.226-3.272) | 0.028 | 1.000 | 2.246(1.092-4.620) | 0.010 | 0.640 | 1.787(1.146-2.788) |
| rs7916582 | T/C | 0.194 | 1.000 | 1.380(0.849-2.244) | 0.119 | 1.000 | 1.318(0.932-1.866) | 0.124 | 1.000 | 1.489(0.896-2.473) | 0.070 | 1.000 | 1.326(0.977-1.801) |
|  | C/C | 0.264 | 1.000 | 2.431(0.512-11.539) | 0.874 | 1.000 | 0.905(0.264-3.108) | 0.121 | 1.000 | 3.055(0.744-12.540) | 0.385 | 1.000 | 0.593(0.182-1.928) |

a. *P* values from unconditional logistic regression analyses, adjusted for age and gender.

b. *P_adjusted_*, *P* value with Bonferroni correction, *P_adjusted_* value less than 0.05 was considered to be significant.

c. OR, Odds Ratio; CI, confidence interval.

-, not available because of the rarity of genotype.
