# Supplementary material for: Mannose-binding lectin 2 gene polymorphisms and their association with tuberculosis in a Chinese population
Source: Infect Dis Poverty. 2020 Apr 29;9:46. doi: 10.1186/s40249-020-00664-9 (PMC7191747; doi:10.1186/s40249-020-00664-9)
Supplement: Supplementary file 4 — Additional file 4: Table S4. Association analysis of MBL2 SNPs between TB subgroups and healthy controls under a dominant and recessive genetic model. [file 40249_2020_664_MOESM4_ESM.docx]

**Table S4 Association analysis of *MBL2* SNPs between TB subgroups and healthy controls under the a dominant and recessive genetic model.**

| **SNP** | **Genetic models** | **EPTB** | | | **PTB** | | | **PTB+EPTB** | | | **TPTB** | | |
| --- | --- | --- | --- | --- | --- | --- | --- | --- | --- | --- | --- | --- | --- |
|  |  | ***P* ^a^** | ***P_adjusted_* ^b^** | **OR [95% CI] ^c^** | ***P*** | ***P_adjusted_*** | **OR [95% CI]** | ***P*** | ***P_adjusted_*** | **OR [95% CI]** | ***P*** | ***P_adjusted_*** | **OR [95% CI]** |
| rs2099902 | (C/T+C/C) vs T/T | 0.035 | 1.000 | 1.543(1.031-2.310) | 0.025 | 1.000 | 1.389(1.043-1.851) | 0.170 | 1.000 | 1.348(0.880-2.063) | 0.002 | 0.128 | 1.500(1.165-1.931) |
|  | C/C vs (T/T+C/T) | 0.070 | 1.000 | 2.159(0.940-4.961) | 0.039 | 1.000 | 2.039(1.037-4.010) | 0.896 | 1.000 | 0.933(0.331-2.633) | 0.015 | 0.960 | 2.125(1.158-3.898) |
| rs930507 | (C/G+G/G) vs C/C | 0.020 | 1.000 | 1.638(1.082-2.480) | 0.004 | 0.256 | 1.525(1.141-2.038) | 0.067 | 1.000 | 1.490(0.972-2.283) | 0.001 | 0.064 | 1.524(1.179-1.969) |
|  | G/G vs (C/C+C/G) | 0.276 | 1.000 | 0.486(0.133-1.779) | 0.339 | 1.000 | 1.403(0.701-2.807) | 0.464 | 1.000 | 1.457(0.532-3.988) | 0.237 | 1.000 | 1.449(0.784-2.678) |
| rs10824793 | (G/A+G/G) vs A/A | 0.079 | 1.000 | 1.427(0.959-2.123) | 0.002 | 0.128 | 1.564(1.180-2.073) | 0.006 | 0.384 | 1.821(1.191-2.783) | 0.001 | 0.064 | 1.516(1.184-1.940) |
|  | G/G vs (A/A+G/A) | 0.490 | 1.000 | 1.281(0.635-2.585) | 0.034 | 1.000 | 1.662(1.040-2.654) | 0.120 | 1.000 | 1.719(0.869-3.399) | 0.067 | 1.000 | 1.489(0.972-2.280) |
| rs7916582 | (T/C+C/C) vs T/T | 0.138 | 1.000 | 1.431(0.892-2.296) | 0.143 | 1.000 | 1.288(0.918-1.808) | 0.063 | 1.000 | 1.587(0.975-2.584) | 0.114 | 1.000 | 1.273(0.944-1.716) |
|  | C/C vs (T/T+T/C) | 0.303 | 1.000 | 2.259(0.479-10.659) | 0.804 | 1.000 | 0.856(0.250-2.929) | 0.150 | 1.000 | 2.807(0.688-11.458) | 0.333 | 1.000 | 0.560(0.173-1.815) |

a. *P* values from unconditional logistic regression analyses, adjusted for age and gender.

b. ***P_adjusted_***, *P* value with Bonferroni correction, *P_adjusted_* value less than 0.05 was considered to be significant.

c. OR, Odds Ratio; CI, confidence interval.
